# Polymorphic Ring-Shaped Molecular Clusters Made of Shape-Variable Building Blocks

**DOI:** 10.3390/nano5010208

**Published:** 2015-02-16

**Authors:** Keitel Cervantes-Salguero, Shogo Hamada, Shin-ichiro M. Nomura, Satoshi Murata

**Affiliations:** 1 Department of Bioengineering and Robotics, Tohoku University, Sendai 980-8579, Japan; E-Mails: cervantes@molbot.mech.tohoku.ac.jp (K.C.-S.); nomura@molbot.mech.tohoku.ac.jp (S.M.N.); 2 Kavli Institute at Cornell for Nanoscale Science, Cornell University, Ithaca, NY 14853, USA; E-Mail: sh964@cornell.edu

**Keywords:** DNA nanostructure, DNA origami, DNA stacking, reconfiguration, substrate

## Abstract

Self-assembling molecular building blocks able to dynamically change their shapes, is a concept that would offer a route to reconfigurable systems. Although simulation studies predict novel properties useful for applications in diverse fields, such kinds of building blocks, have not been implemented thus far with molecules. Here, we report shape-variable building blocks fabricated by DNA self-assembly. Blocks are movable enough to undergo shape transitions along geometrical ranges. Blocks connect to each other and assemble into polymorphic ring-shaped clusters via the stacking of DNA blunt-ends. Reconfiguration of the polymorphic clusters is achieved by the surface diffusion on mica substrate in response to a monovalent salt concentration. This work could inspire novel reconfigurable self-assembling systems for applications in molecular robotics.

## 1. Introduction

Structures that can change their shapes are of interest at all scales, not only for the potential applications, but also because the design principles may be used across scales [1,2,3,4,5,6,7,8]. Switching structural dimensions [4] and topology [5], and partial detachment/attachment [7] of individual nanostructures, as well as controlling the number of components through geometric complementarity [1,8] and specific logic rules for targeting a configuration [2,3] of interacting macrostructures, are among some approaches for shape-reconfiguration. Of particular interest is the metamorphosis of clusters of mechatronic modules [3], where incorporated degrees-of-freedom allowed shape transitions in each module, generating locomotion and reconfiguration of the modules as a whole. However, there is still a bridge to be crossed between the programmable dynamic behaviour of individual molecular systems and the programmable collective behaviour of top-down fabricated structures. Indeed, the bottom-up fabrication of reconfigurable assemblies of interacting molecular robots remains challenging [9]. Towards this goal, we take a direct approach by designing building blocks with movable parts and argue that the shape properties of these blocks govern their assembly.

The shape properties of building blocks is important in molecular self-assembly, where the shape of a nanostructure or colloidal particle is defined as the surface geometry and the interaction field around the particle surface [10,11]. Although there has been extensive study of static particles [12,13,14,15,16], only a few contributions exist on the fabrication of structures with dynamic shapes [17,18,19,20]. Nevertheless, the work on micro- and nano-reconfigurable structures has recently inspired different simulation studies for the assembly and reconfiguration of assemblies of shape-changing nanorods, shape-shifting blocks, patchy nanoparticles with dynamic covalent bonds, and flexible lock-and-key colloids [21,22,23,24,25]. Although these simulations predict notable properties, such as switchable band gaps for optical devices or tunable pores for drug delivery, the field is open for actual molecular structures with variable shapes. Recently, complaint nanostructures made of DNA were demonstrated [26]. In our work, shape-variable building blocks are realized by harnessing the properties of DNA.

DNA does not only plays important roles in molecular biology, but it has also been used to fabricate nanostructures of various shapes and functionalities. Nanostructures ranging from molecular walkers on programmable tracks to static closed topologies, and from pre-stressed tensegrity structures to reconfigurable structures [4,5,17,26,27,28,29,30,31,32,33,34,35]. Undoubtedly, DNA has a great potential for engineering novel applications due to its programmability, rigidity, and flexibility [6,36,37]. Here, we show the concept, design, and experimentation of shape-variable building blocks made of DNA that are capable of self-assembly into polymorphic closed clusters. Our experimental setup provides a platform for studying the cluster disassembly and re-assembly (reconfiguration) on substrate in response to a monovalent salt concentration.

## 2. Experimental Section

**Figure 1:**
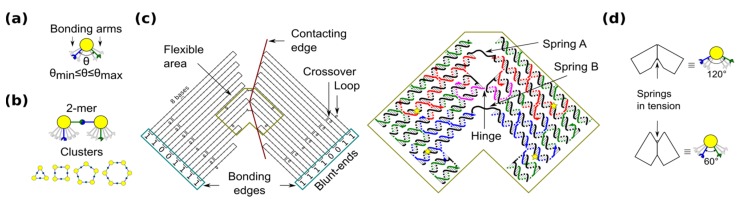
Shape-variable building block and design with DNA origami. (**a**) Abstract representation of the block with two bonding arms; (**b**) 2-mers in *cis* configuration and assembled polymorphic clusters (from 3-mers up to 6-mers); (**c**) (**left**): Origami scaffold (black), flexible area (mustard), contacting edges (red) and blunt-ends with binary codes (sky-blue); (**right**): Detail of the flexible area showing staples in colour (yellow stars indicate scaffold loops for tuning springs); (**d**) Origami profile and its correspondent abstract representation for the 60° and 120° configurations.

The shape-variable building blocks (also called monomers) are restricted to homogenous blocks which self-assemble into clusters (also called *x*-mers) with finite size or limited number of building blocks (*i.e.*, *x*). The characteristics of the blocks are inspired by the flexibility of 3-point star DNA tiles [17], which allows the formation of closed nanostructures. Among closed topologies that have been used for DNA nanostructures [17,27,28,29], a ring is the simplest case because component monomers require only two bonding arms. In our blocks, the angle between bonding arms can adopt any value between a minimum and maximum (Figure 1a). These arms have complementary bonding edges and make bonds to each other in such a way that a 2-mers makes a *cis* configuration (Figure 1b, top). This angle implicitly defines the number of monomers in a cluster, namely the size of the self-assembled ring (Figure 1b, bottom).

The shape-variable building blocks are fabricated with the DNA origami technique [35]. The block is a DNA origami with two symmetrical parts that resembles the two bonding arms explained above. The edge of each arm consists of DNA blunt-ends of which stacking interaction strength can be programmed and it is represented with a binary code [31]. The block incorporates: (1) a flexible area (enclosed area in Figure 1c), which provides degrees-of-freedom to the block; and (2) contacting edges, which set two angular limits of 60° and 120° (Figure 1d). In the flexible area, the block arms are linked by means of a single phosphate of a staple strand (a hinge that allows rotation) and two unpaired scaffold segments. These scaffold segments have two functions. One function is to prevent undesired degrees-of-freedom, such as relative twist of the block arms. Another function of the segments is to act as entropic springs (spring A and B) that tend to move back to states with higher entropy when stretched. The nominal length of each spring can be tuned by spooling the scaffold forming loops in the origami (Figure 1c, yellow stars), similarly as done for tuning the tension in tensegrity structures [30]. For example, by exchanging the blue staples in Figure 1c, it is possible to adjust the nominal length of spring B. DNA sequences of the springs should not form secondary structures and should be flexible. These sequences are chosen along the DNA scaffold in such a way that five thymines are kept in the middle of each spring (Supplementary Information S1). Details of the staples and DNA sequences are shown in Supplementary Information S13.

For simplicity, we define the notation *M* (*a*,*b*) for the monomer with a (spring A) and b (spring B) nucleotides (nt). *a* = 11 nt and *b* = 8 − 18 nt are set in accordance with the flexibility of single stranded DNA (Supplementary Information S2). Monomers are prepared in a solution containing 1 × TAE/Mg^2+^ (40 mM Tris, 20 mM acetic acid, 2 mM EDTA, 12.5 mM Mg acetate), heated and cooled down (Supplementary Information S3).

## 3. Results and Discussion

**Figure 2:**
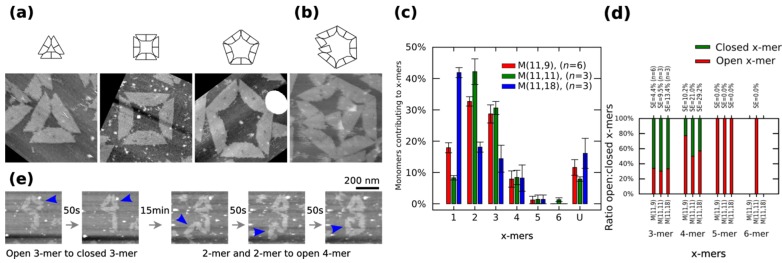
Polymorphic clusters made of shape-variable monomers before “reconfiguration protocol” (**a**–**d**) and during the “reconfiguration protocol” (**e**). (**a**–**b**) Clusters of *M* (11,11). First row: cluster representations. Second row: AFM images. 3-mers, 4-mers and 5-mers (**a**) and open 6-mers (**b**) at 2 nM concentration. (**c**–**d**) Distribution of monomers contributing to the formation of *x*-mers in open and closed states (**c**) and ratio open:closed *x*-mers (**d**) for *M* (11,9), *M* (11,11) and *M* (11,18). *U* indicates unclear monomers. (**e**) Inset of the frames in movie in Supplementary Information (0.02 fps). A 3-mers closes and two 2-mers self-assemble into an open 4-mer. AFM images in (**a**) are 310 nm × 300 nm in size. Error bars in (**c**) and SE in (**d**) indicate the standard error. *n* indicates the number of analysed AFM images of 2040 nm × 1680 nm, and the number of counted monomers per each AFM image is shown in Figure S12.

We characterize the monomers on mica substrate by using atomic force microscopy (AFM) under 1 × TAE/Mg^2+^, which is basically practically enough to keep the origami immobilized (Figure S12). AFM images indicate the formation of polymorphic clusters out of *M* (11,11). Clusters can be in a closed or open state. Figure 2a shows closed clusters, and Figure 2b shows an open 6-mers (no closed 6-mers is found in our AFM observations). The cluster size distribution, the number of monomers contributing to each particular cluster, is obtained by calculating the normalized number of *x*-mers, in an open or closed state, among several AFM images and multiplying it to the number of monomers in the *x*-mer. The distribution of 1-mers, 2-mers, and 3-mers changes when varying the length of spring B to 9 nt or 18 nt (Figure 2c). For a given x-mer, we calculate the *p*-values for those springs (Supplementary Information S12.2). For the analysed springs, 1-mers show a low value at 11 nt (*p* < 0.01) and a high value at 18 nt (*p* < 0.01). 2-mers and 3-mers show a low value at 18 nt (*p* < 0.01 and *p* < 0.05, respectively). An increment of 1-mers is consistent with the idea that a greater spring B gives more flexibility to the monomer and makes the connection with other monomer more difficult, which leads to less 2-mers and as a consequence less 3-mers. A variation in the distribution of 2-mers is also consistent with transition state theory, where the reaction between two particles is described as the relative orientations between them; here, although the surface area of reaction is constant (bonding edges do not change), a flexible monomer generates a different energy landscape compared to a less flexible one. As expected, increasing spring B to 11 nt increases the ratio closed:open 4-mers (*p* = 0.04) (Figure 2d). There are also some kinetic barriers for the formation of *x*-mers as found by the parallel polarity (Supplementary Information S8.1.4), mismatching and dislocation (Figure S13) of stacking bonds. Closed dimers (Supplementary Information S8.1.2) and larger clusters (*x* > 6; Supplementary Information S8.1.5) are also found. We identify the type of clusters after deposition based on the observation of those kinetic barriers (Supplementary Information S8.1.6).

Self-assembled clusters on mica surface can reconfigure in response to monovalent cations. Monovalent cations, such as NaCl are known to reduce the DNA-mica binding interaction [38], causing diffusion of DNA origami on mica substrate [39,40]. A 1 × TAE/Mg^2+^ buffer solution containing 100 mM NaCl is put on top of mica with *M* (11,11) for four hours (this protocol is called “reconfiguration protocol” hereafter, also in Supplementary Information S6). Then nanostructures are observed by AFM under 1 × TAE/Mg^2+^ buffer solution containing 100 mM NaCl. As a result, monomers and clusters are observed diffusing and re-self-assembling on mica surface (movie in Supplementary Information). Figure 2e shows an inset of the movie. The connectivity between shape-variable monomers and the distribution on mica of the monomers do change after the “reconfiguration protocol”: long polymers as well as areas with high populations of monomers appear (Supplementary Information S8.2).

**Figure 3:**
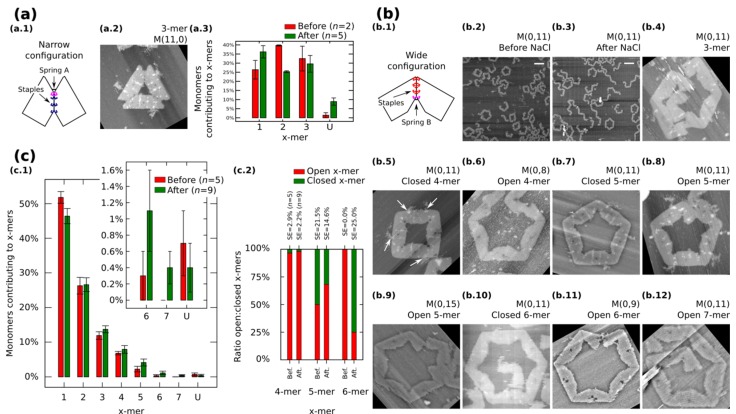
Self-assemble and reconfiguration of fixed monomers. (**a.1**) and (**b.1**): Fixed monomers. (**a.2–3**) 3-mers and cluster distribution before and after reconfiguration of *M* (11,0). (**b.2–3**) Representative AFM images of M (0,11) before and after reconfiguration. (**b.4–12**) Clusters after reconfiguration for different spring B. White arrows in (**b.5**) show extra M13. (**c**): Distribution of monomers contributing to the formation of *x*-mers including open and closed states (**c.1**) and ratio open:closed *x*-mers (**c.2**) for M (0,11). U indicates unclear monomers. AFM images are 310 nm × 300 nm. Width of (**b.9**) is 500 nm. Scale bars are 200 nm. Error bars in (**a.3, c.1**) and SE in (**c.2**) indicate the standard error. *n* indicates the number of analysed AFM images of 2040 nm × 1680 nm, and the number of counted monomers per each AFM image is shown in Figure S12.

We also restrict our building blocks to fixed shapes. Fixed monomers are prepared in one-pot reaction by bridging the contacting edges with additional staples (Figure 3a.1 and Figure 3b.1). *M* (*a*,*b* = 0) (Figure 3a.1) and *M* (*a* = 0,*b*) (Figure 3b.1) denote fixed monomers in wide and narrow configuration, respectively.

Fixed monomers *M* (11,0) self-assemble into 3-mers. AFM after sample deposition shows as much as 32.5% of monomers forming 3-mers (Figure 3a.3 and Figure S9). After the “reconfiguration protocol” the shape of histogram changes indicating a decrement of 2-mers (*p* < 0.01).

AFM images of *M* (0,11) prepared in solution show the formation of polymorphic clusters (Figure 3b.2). 4-mers (28 open and one closed) and 5-mers (four open and four closed) are found in open and closed states among five AFM images of 2040 nm × 1680 nm (number of monomers per image are indicated in Figure S12). However, only one open 6-mers is observed (among 179 monomers in one AFM image), indicating kinetic traps in the formation process of 6-mers. These traps are possibly due to the flexibility of the origami, in other words, the stacking interaction compensates the energy required to bend the DNA helices favoring narrow angles for the monomer.

*M* (0,11) on mica is prepared by following the “reconfiguration protocol” and observed by AFM under 1 × TAE/Mg^2+^ (Figure 3b.3). Figure 3c.1 shows the distribution of monomers contributing to each particular cluster in open and closed states. In general, small clusters have a high yield and no significant difference exists before and after addition of NaCl except for 1-mers (*p* < 0.05) and the appearance of 7-mers (Figure 3b.12; 0.4% with SE = 0.2%). The ratio of open:closed clusters shows the dominance of closed 6-mers after the addition of NaCl (Figure 3c.2; from 0.0% to 75% with SE = 25%). We can speculate two causes for the formation of closed 6-mers, (1) the diffusion and fluctuation of the origami is restricted to the surface favouring on-plane connections with neighbouring monomers; and (2) the concentration of NaCl in the buffer enhances the electrostatic screening between the arms of the monomer and consequently allowing a wider angle for assembling 6-mers. White arrows in Figure 3b.5 indicate the extra M13 of the origami. This extra M13 suggests connections through parallel stacking polarity (Supplementary Information S8.1.4).

In order to explore the effect of the springs, we tune the length of the spring B and apply the “reconfiguration protocol”. Figure 3b.4−12 show representative clusters for different spring B. In the case of *M* (0,9), monomers in 6-mers show stretched strands and angles less than the designed 120° (Figure 3b.11). We say these clusters are open 6-mers.

**Figure 4:**
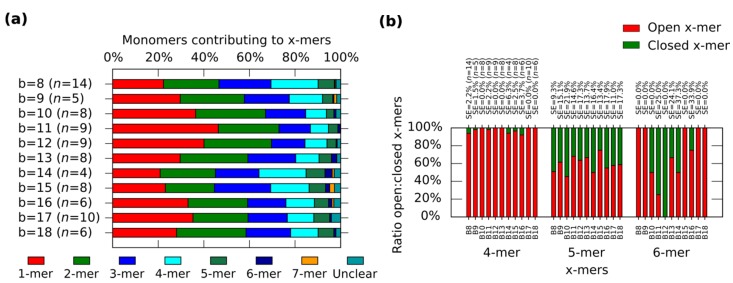
Distributions of the clusters after reconfiguration for *M* (0,*b*) (*b* = 8−18). (**a**) Distribution of monomers contributing to the formation of *x*-mers including open and closed states. Colours indicate each type of cluster. Yellow indicates open 7-mer. Analysed AFM areas are 2240 nm × 1680 nm; (**b**) Ratio open:closed clusters for 4-mers, 5-mers and 6-mers. Error bars in (**a**) and SE in (**b**) indicate the standard error. *n* indicates the number of analysed AFM images of 2040 nm × 1680 nm, and the number of counted monomers per each AFM image is shown in Figure S12.

In general, the distribution shows the tendency for forming clusters with yields varying according to the spring length (Figure 4a). For a given *x*-mer, we find critical springs that show significant difference in the yields (Supplementary Information S12.2). For 1-mers, critical springs with 8 nt, 12 nt, 15 nt, 17 nt and 18nt (*p* < 0.01). For 2-mers, critical springs with 8 nt, 13 nt, 15 nt and 18 nt (*p* < 0.01). For 3-mers, critical springs with 8 nt, 11 nt, and 15 nt (*p* < 0.01). For 4-mers, critical springs with 8 nt, 11 nt, 15 nt, and 17 nt (*p* < 0.01). For 5-mers, critical springs with 8 nt and 10 nt (*p* < 0.01). Critical springs with 8 nt and 13 nt for 6-mers (*p* < 0.05), and critical springs with 10 nt, 15 nt and 18 nt for 7-mers (*p* < 0.05). Varying the spring length does not only affect the yield of *x*-mers, but also the tendency for closed states (Figure 4b). We also find critical springs for the ratio open:closed clusters (Supplementary Information S12.2). 8 nt (*p* < 0.01) and 16 nt (*p* < 0.05) show a high ratio closed:open 4-mers when comparing with near springs. For 12 nt all 6-mers are closed (one 6-mers among 276 monomer, and other 6-mers among 334 monomers from nine AFM images of 2040 nm × 1680 nm), and for springs greater than 11 nt or 12 nt, the number of closed 6-mers seem to decrease (*p* < 0.05). We explain these results considering that short springs pull the bonding arms; and, as a result, the formation of one type of cluster is favoured over the formation of other types, as shown by the critical spring with 11 nt. In addition, closed 4-mers form if the angle of each monomer approximates 90°, which happens if the spring is short, as with the critical spring with 8 nt. The critical spring with 8 nt is comparable with our calculated minimum spring length of 9 nt (Supplementary Information S2). On the other hand, if the spring is longer, the monomer becomes more flexible and it may be difficult for the cluster to close, as shown by the critical springs of 11 nt and 12 nt. These results indicate that the stiffness can be adjusted by tuning the spring length.

The formation of the clusters is time dependent (Supplementary Information S11). Sample *M* (11,9) is at room temperature for one month and nanostructures are observed by AFM. As a result, the distribution of *M* (11,9) changes. A similar behavior occurs with sample *M* (0,11).

## 4. Conclusions

In summary, we have studied the self-assembly and reconfiguration of clusters made of shape-variable building blocks. We found that the cluster formation depends on the block shape. Moreover, we found that geometric constrains, as well as kinetic barriers, inhibit the formation of large clusters. It may not be difficult to expand the variety of shape-variable blocks for assembling other clusters and crystals, but some issues, such as the degree of flexibility of the block, should be addressed carefully. Finally, the shape-variable building blocks have provided a platform for the implemention of those scenarios proposed in the literatures for reconfiguring molecular assemblies.

## Supplementary Materials

Supplementary materials can be accessed at: http://www.mdpi.com/2079-4991/5/1/208/s1.
