# Supplementary material for: Polymorphic Ring-Shaped Molecular Clusters Made of Shape-Variable Building Blocks
Source: Nanomaterials (Basel). 2015 Feb 16;5(1):208–17. doi: 10.3390/nano5010208 (PMC5312864; doi:10.3390/nano5010208)
Supplement: Supplementary file 1 [file nanomaterials-05-00208-s001.pdf]

Supplementary Information

S1. Springs Sequences

- Spring A = … T A T T A T T T T T C T T G T … = *a* bases
- Spring B = … C C T G A T T T T T G A T T T … = *b* bases

S2. DNA Backbone

A single-stranded DNA behaves as a flexible and extensible polymer depending on the force exerted on it [1]. This behavior is due to the molecular structure of the sugars in the backbone. There are two pucker configurations for the sugars: C3' and C2'. In a C2' pucker configuration the inter-phosphate distance becomes 0.7 nm [2]. This distance (0.7 nm) is comparable to the inter-base pair distance of double-stranded DNA in B-form (0.32 nm).

We roughly calculate the straight distance of spring B at $b = 0$ and $a = 0$ conditions and divide it by a length of 1 nt to calculate a critical value; in reality, ssDNA behaves as entropic springs, so the actual critical value might become larger than this estimation, however, we simply use this value as a minimum.

Numerically the straight distance we obtain is 7.48 nm (spring A) and 6.28 nm (spring B), for an inter-base-pair distance of 0.34 nm. If we consider that the inter-phosphate distance for a C2' endo pucker configuration we obtain 11 nt and 9 nt for spring A and B, respectively. An inter-phosphate distance for a C3' endo pucker configuration (0.59 nm) gives 13 nt and 11 nt for spring A and B, respectively.

S3. Protocol for Preparation of DNA Origami

100 µL of DNA origami is prepared by mixing 2 nM of M13mp18 (scaffold) with 75 nM of staple strands in 40 mM Tris, 20 mM acetic acid, 2 mM EDTA, and 12.5 mM Mg acetate (1 × TAE/Mg^{2+}). The mixture is heated up and kept at 90 °C for 5 min, then it is cooled down to 4 °C at a rate of −1 °C/min. Samples are stored at 4 °C. Samples for AFM observation are left at room temperature for more than 4 h and then used. In order to double the concentration of monomers, the concentration of scaffold is 4 nM with 100 nM of staple strands.

S4. Protocol for AFM Observation

We characterize the monomers on mica substrate by using atomic force microscopy (AFM) under 1 × TAE/Mg^{2+}, which is basically enough to the keep the origami immobilized. The protocol consists of the following steps:

1. 2 µL of sample is deposited on a freshly cleaved mica substrate of 1.5 mm diameter (a few times with 2 mm diameter) for different deposition times.
   - The usual deposition time for shape-variable monomers is 10 min.
   - The usual deposition time for fixed monomers is 25 min.
2. Then samples are rinsed with 10 µL of 1 × TAE/Mg^{2+}, leaving a small drop on top of mica.
3. Samples are observed using AFM under 1 × TAE/Mg^{2+} buffer.

S5. Reconfiguration Method

The reconfiguration is described in four steps (Figure S1):

1. The polymorphic clusters are self-assembled in solution (3D).
2. Structures are deposited on a 2D surface.
3. Structures are diffused on the 2D surface.
4. Shape-variable monomers diffuse and eventually self-assemble into new polymorphic clusters.

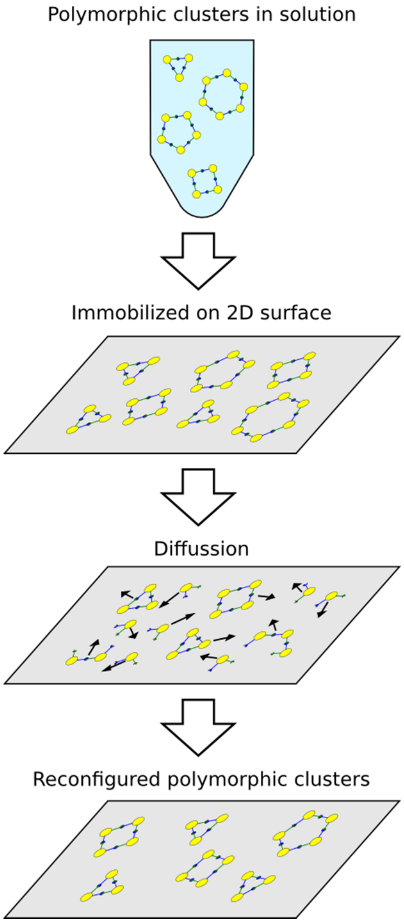

Figure S1. Clusters diffusion and disassembly on 2D surface.

Polymorphic clusters diffuse on the 2D surface, disassemble and self-assemble again into new clusters. In other words, the reconfiguration can be regarded as a re-self-assembly of shape-variable monomers.

S6. Reconfiguration Protocol

Samples previously observed by AFM are prepared as follows:

1. Mica sample is taken directly from the AFM (we realize that the surface of mica containes a layer of buffer on top, then it seems to be a good practice to blow the surface with air before applying the following step).
2. 1 µL (this value is optimized because 2 µL drops sometimes fall off of the mica) of 1 × TAE/Mg^{2+} buffer containing 100 mM NaCl is put on top of mica for about 4 h (this is an arbitrary value).

3. Samples are observed using AFM under 1 × TAE/Mg^{2+} buffer (it is expected that the drop containing NaCl on top of the mica diffuses through the AFM observation buffer).

Samples, which are not observed previously by AFM are prepared as follows:

1. 2 μL of sample is deposited on a freshly cleaved mica substrate of 1.5 mm diameter for different deposition times.
2. Then sample is rinsed with 10 μL of 1 × TAE/Mg^{2+}, leaving a drop on top of the mica. The mica surface is blown with air.
3. 1 μL of 1 × TAE/Mg^{2+} buffer containing 100 mM NaCl is put on top of mica for about 4 h (this is an arbitrary value).
4. Sample is observed using AFM under 1 × TAE/Mg^{2+} buffer.

Sample for the movie is prepared the same way as the "reconfiguration protocol" with the exception that the AFM observation is done under a 1 × TAE/Mg^{2+} buffer solution containing 100 mM Na^+. The movie is captured at 0.02 fps.

S7. AFM Images Processing

All samples are observed using a high-speed AFM (Nano Live Vision, RIBM, Tsukuba, Japan) with silicon nitride cantilevers (Micro Cantilever BL-AC10DS, BL-AC10FS and BL-AC10EGS, Olympus, Tokyo, Japan). In general, Brightness/Contrast adjustment and Fit Polynomial filter are applied to raw AFM pictures by using ImageJ. A band pass filter is applied when needed.

S8. AFM of Shape-Variable Monomers

S8.1. Immobilization on Mica

Figure S2 shows a representative image of the *M* (11,11) after deposition on mica.

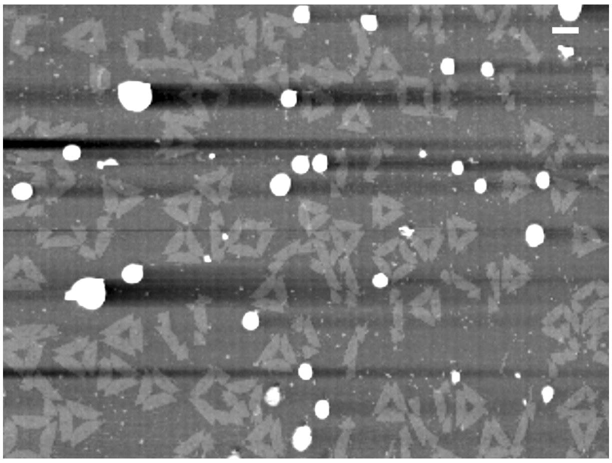

Figure S2. Representative AFM image of the polymorphic clusters made of *M* (11,11). Scale bar is 100 nm.

S8.1.1. Flipping of Bonding Arms

The orientation of the bonding arms can flip (Figure S3). Hereafter, the profiles of the monomers are indicated in blue, red, or green. A flipped arm is indicated with a filled color (blue, red, or green). Cases of flipped arms are not quantified in this work.

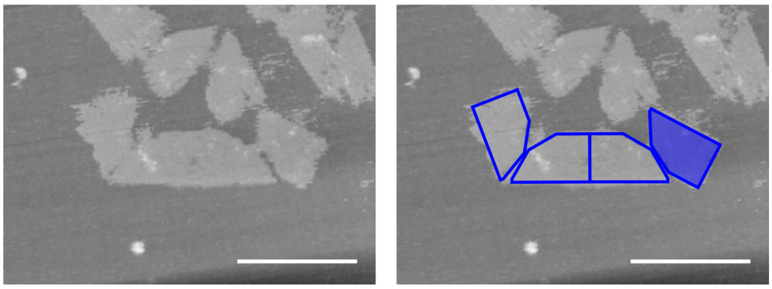

Figure S3. AFM image showing a dimer with a flipped bonding arm (*M* (11,11) at 4 mM with 0.5 min deposition time). Scale bars are 100 nm.

S8.1.2. Closed Dimers

Self-assembled dimers also form closed structures as shown in Figure S4. This structure represents as much as 1.0% for *M* (11,9), 3.5% for *M* (11,11) and 0.0% for *M* (11,18), and 0.0% for *M* (11,9) after a long time at room temperature (see also Supplementary Information S11).

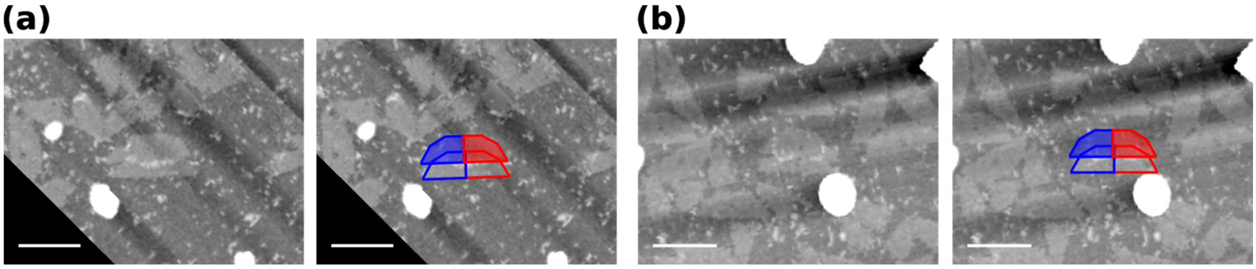

Figure S4. Closed dimers (*M* (11,11)). Scale bars are 100 nm.

S8.1.3. Doubling the Concentration of M13 for *M* (11,11)

The concentration of sample *M* (11,11) is doubled to 4 nM of M13 according to protocol in Supplementary Information S4. A representative image is shown in Figure S5. The hypothesis for this experiment is to obtain larger clusters in similar manner as for the flexible DNA tile of Chengde Mao. In our experiment, large polymers are formed (Figure S5d).

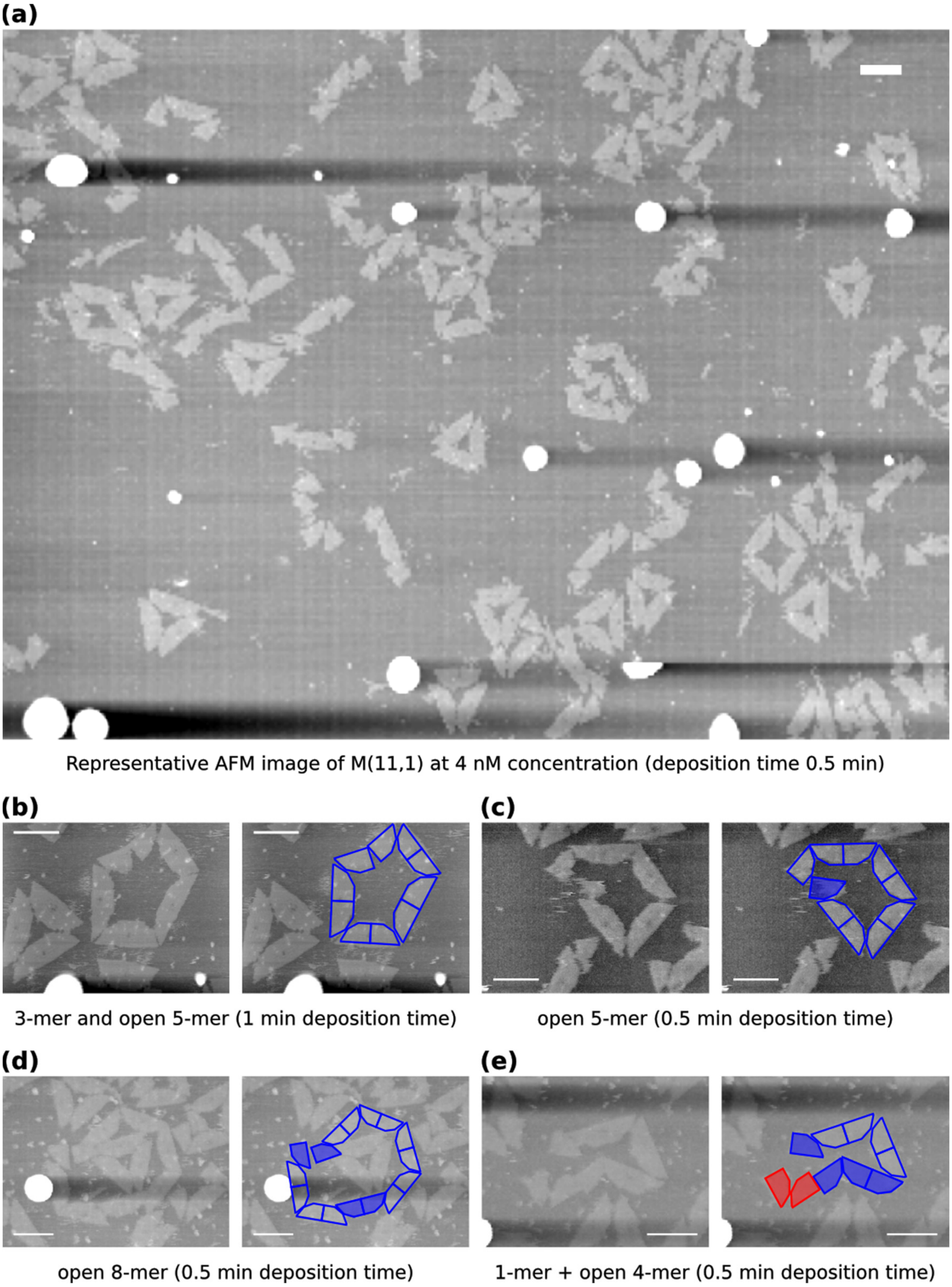

Figure S5. AFM images for double concentration of *M* (11,11). Scale bars are 100 nm.

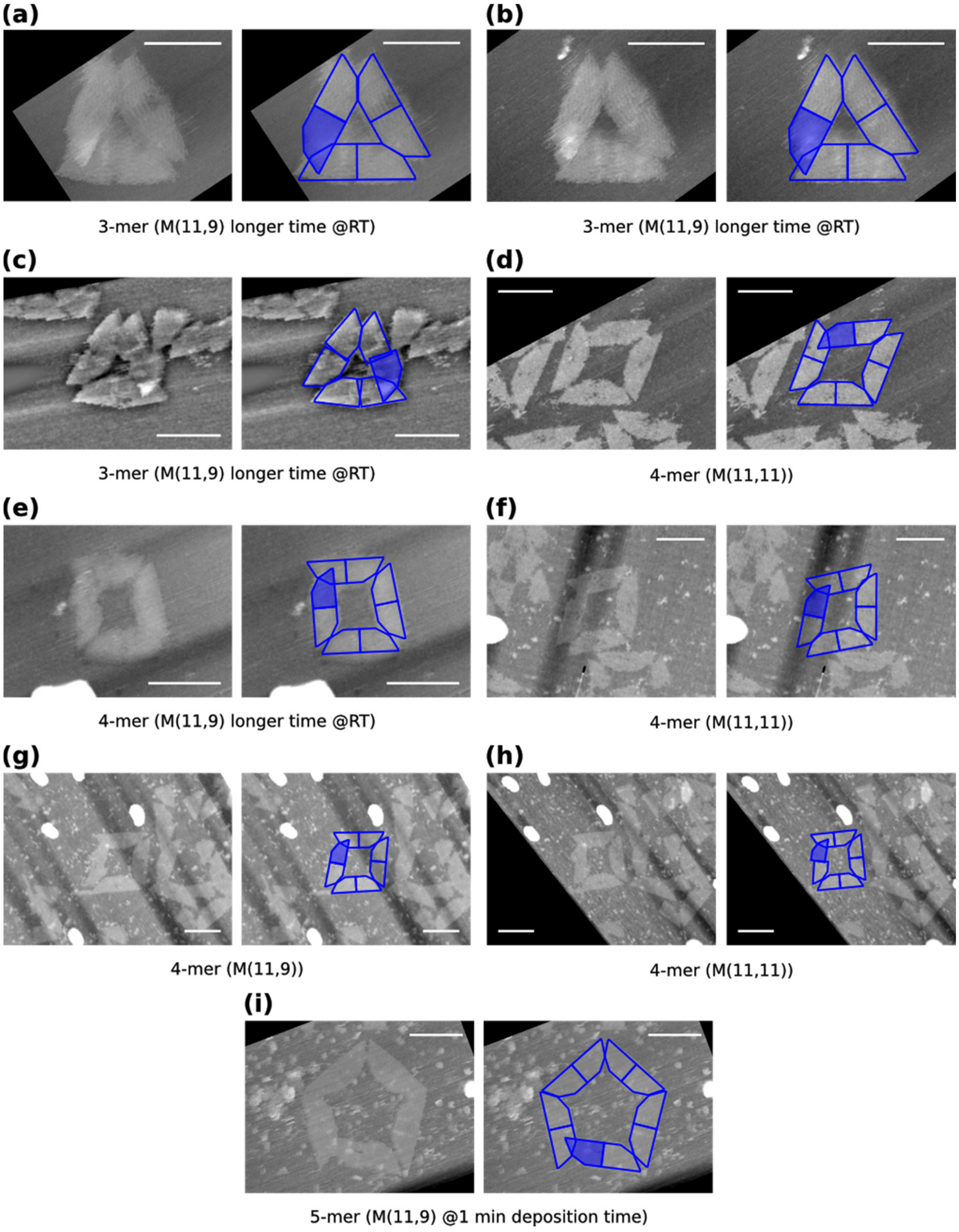

Figure S6. Mismatching of the stacking bonds: parallel polarity (*M* (11,11)). Scale bars are 100 nm.

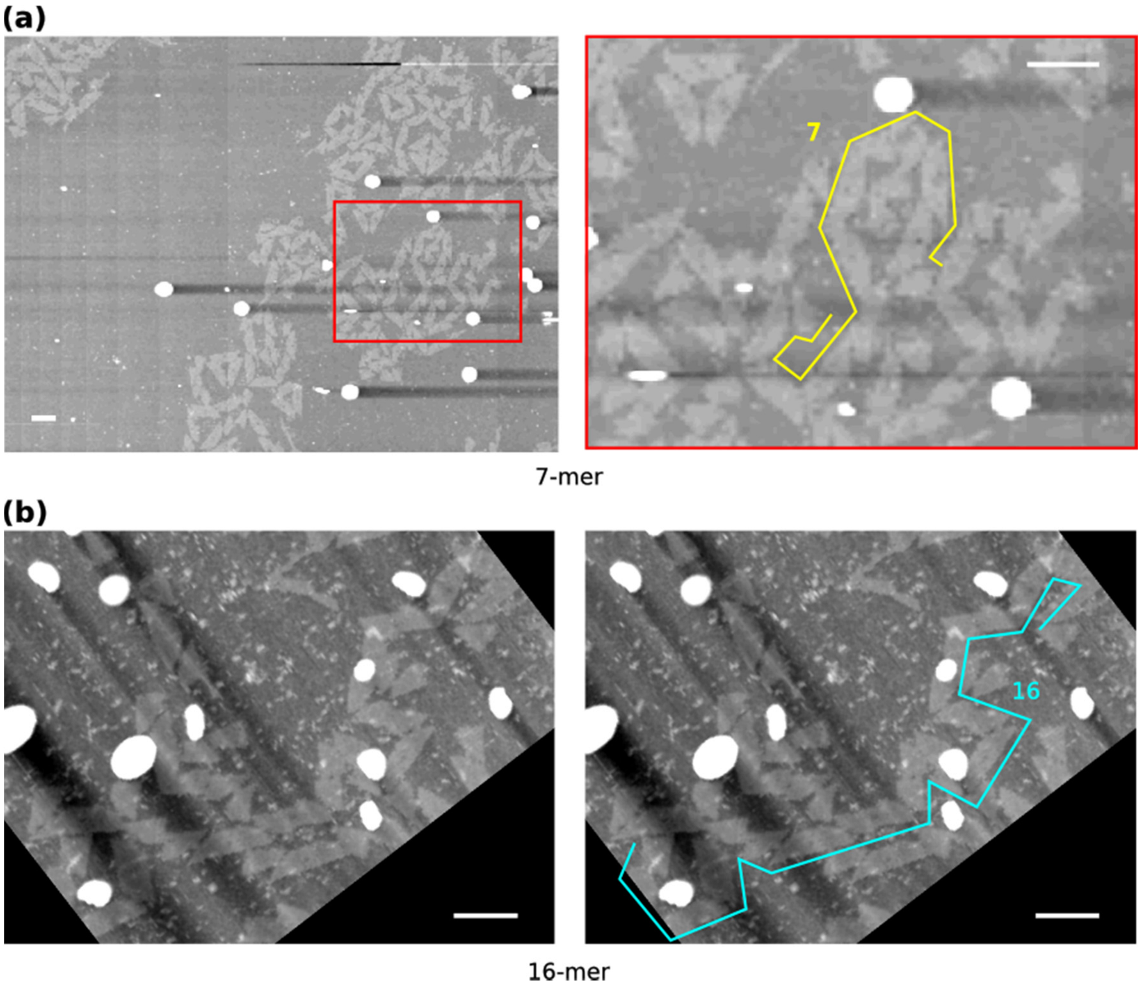

Figure S7. Larger clusters for *M* (11,11) at 2 nM.

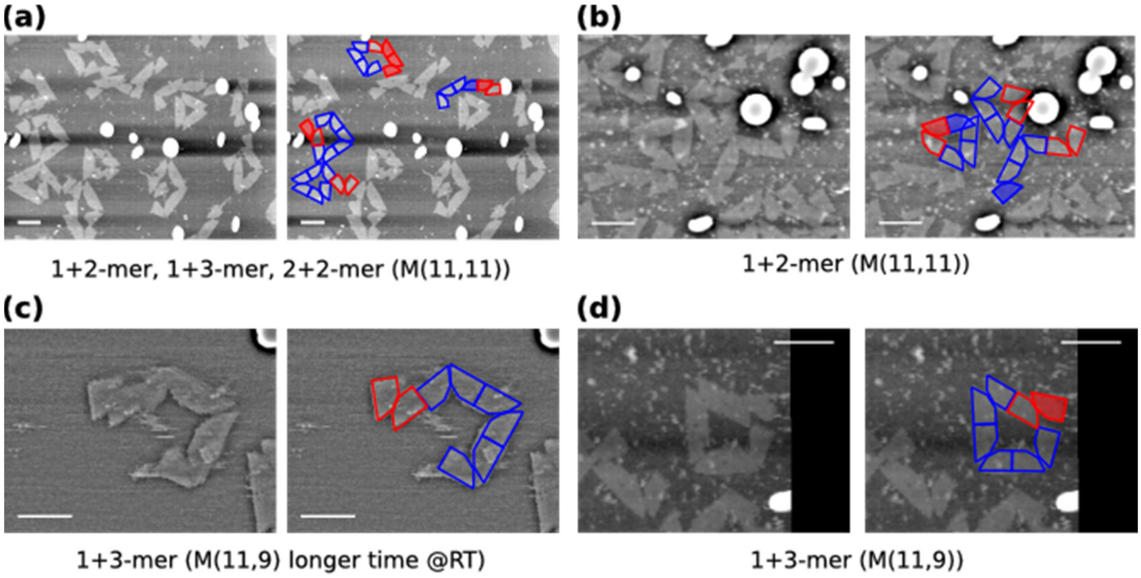

Figure S8. *Cont.*

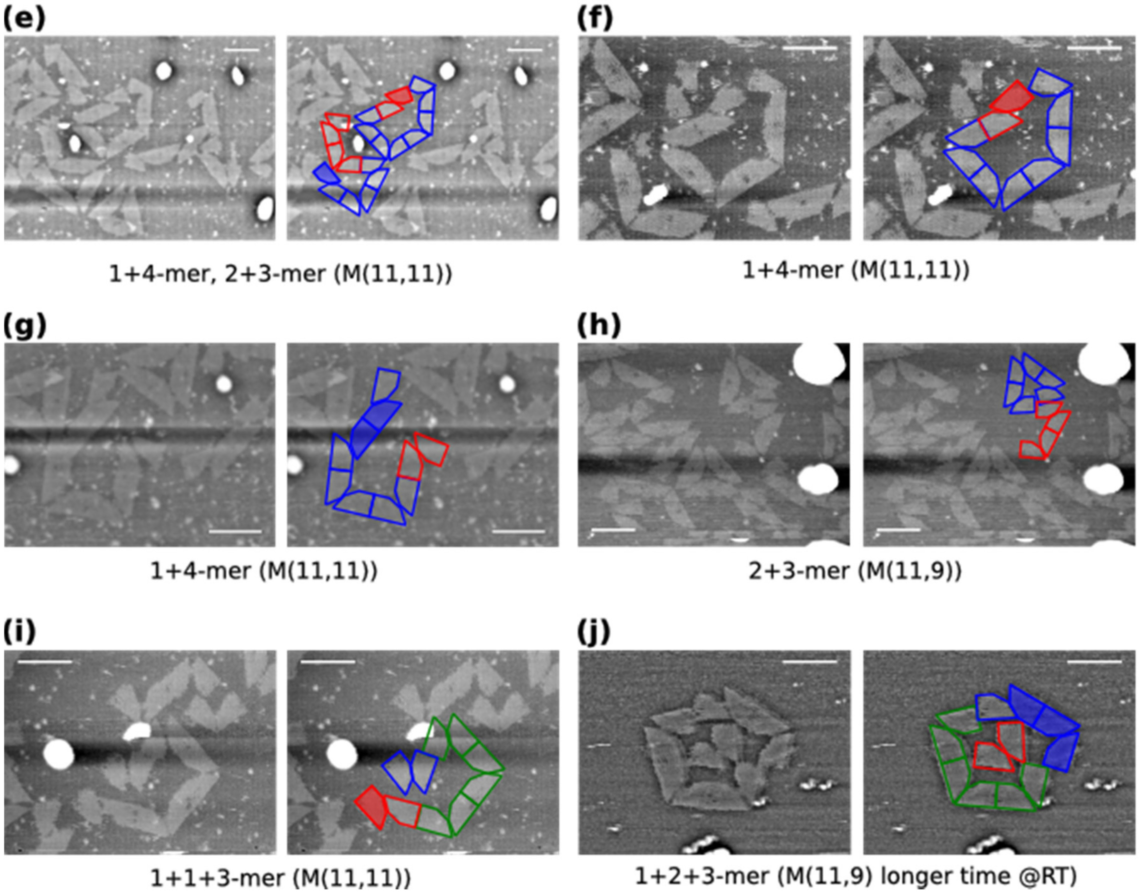

Figure S8. Analysis of clusters presenting mismatching.

S8.1.4. Mismatching: Stacking Bonds with Parallel Polarity

Programmable stacking interactions as described by Woo and Rothemund [4] are based on the assumption that all the minor grooves facing one side of the stacking bond plane create a stacking polarity. Then, regions of minor grooves with antiparallel stacking polarity are supposed to form a bond. In our design this polarity is believed to ensure bonds to be in *cis* configuration while avoiding other configurations. However, our results indicate that stacking bonds can be formed by mismatching. Figure S6 shows stacking bonds with parallel polarity found after deposition on mica (before reconfiguration).

Finding experimental evidence of mismatching of stacking bonds through parallel stacking polarity is an unexpected result. In fact, no experiment thus far has demonstrated parallel polarity as a mean for forming stacking bonds. However, it seems that shape-complementarity stacking bonds can be formed with parallel polarity [4].

Finding bonds with parallel polarity for the shape-variable monomer after deposition on mica can be explained considering the degrees-of-freedom of the monomer. On the other hand, in the original stacking paper *cis-trans* experiments are performed for structures with static shapes. A corner of triangle only needs to match geometrically into a 2-mer for closing the cluster, with only one stacking bond being enough for that purpose. However, for a shape-variable monomer it is required two stacking bonds for closing the cluster is required. While one stacking bond may present the correct stacking polarity, the other may be kinetically trapped in a parallel polarity due to the inherent degrees-of-freedom.

S8.1.5. Larger Clusters

Some larger structures (more than six monomers) are observed at a 2 nM concentration of scaffold (Figure S7). Therefore, it is unclear whether to doubling the concentration of the scaffold may have an effect in the formation of 6-mers or larger clusters.

It may be possible that small clusters are originated after the breakage of larger clusters or polymers upon deposition. These larger clusters seem to be polymers of two or three types of small clusters.

S8.1.6. Identification of the Clusters Deposited on Mica

Although the bonding is designed to be a *cis* configuration, AFM images suggest there are mismatching on the stacking bonds. Cases of mismatching are discussed in Supplementary Information S8.1.4 and Figure S13. Cases of *trans* and parallel polarity configurations are less probable, and indicates kinetic traps. In this work, *trans* configurations are regarded as an indicator of the formation of clusters through the union of two or more open clusters.

Sometimes it is difficult to identify what kind of cluster is present. In Figure S8h it is not clear whether the cluster is an open 5-mer or just two clusters (open 3-mer and 2-mer) in close proximity. Figure S8j shows the interaction between a dimer and a trimer, with a monomer in the center. It is possible that the dimer and trimer did not interact with the monomer in solution, or probably all three structures belong to the same cluster. Therefore, this structure is counted as unclear.

Tables S1–S3 shows the clusters, interpreted as in Figure S8, for the same analyzed areas in the cluster size distributions in Figure 2c (main text). Values in the tables indicate the average number of occurrences in an AFM image with 2240 nm × 1680 nm. Samples in solution at room temperature for a long time shows a particular behavior (Supplementary Information I S11), and are labeled as $t = 1$ month in Tables S1–S3.

Table S1. Average number of clusters presenting mismatching as seen in Figure S8. Samples in solution at room temperature for more than a month are labeled as $t = 1$ month.

Sample\Clusters($X + Y$)	1 + 2	1 + 3	1 + 4	1 + 5	2 + 2	2 + 3	2 + 4	3 + 3	3 + 4	4 + 5
$M(11,18)$	1.9%	0.6%	0.0%	0.0%	0.0%	0.0%	0.0%	0.0%	0.0%	0.0%
$M(11,11)$	2.0%	0.8%	0.0%	0.3%	0.6%	0.1%	0.0%	0.0%	0.1%	0.0%
$M(11,9)$	2.6%	1.3%	0.1%	0.0%	0.5%	0.4%	0.1%	0.2%	0.0%	0.1%
$M(11,9)$ for $t = 1$ month	2.3%	1.2%	0.0%	0.0%	0.0%	0.0%	0.0%	0.0%	0.0%	0.0%

Table S2. Average number of clusters presenting mismatching as seen in Figure S8. Samples in solution at room temperature for more than a month are labeled as $t = 1$ month.

Sample\Clusters($X + Y + Z$)	1 + 1 + 1	1 + 1 + 2	1 + 1 + 3	1 + 2 + 2	1 + 2 + 3	1 + 2 + 3
$M(11,18)$	0.3%	0.0%	0.0%	0.0%	0.0%	0.0%
$M(11,11)$	0.0%	0.4%	0.0%	0.1%	0.1%	0.0%
$M(11,9)$	0.1%	0.6%	0.1%	0.0%	0.2%	0.1%
$M(11,9)$ for $t = 1$ month	0.0%	0.0%	0.0%	0.0%	0.1%	0.0%

Table S3. Average number of clusters presenting mismatching as seen in Figure S8. Samples in solution at room temperature for more than a month are labeled as $t = 1$ month.

Sample\Clusters($X + Y + Z + W$)	1 + 1 + 1 + 1	1 + 1 + 2 + 2
M (11,18)	0.0%	0.3%
M (11,11)	0.0%	0.0%
M (11,9)	0.3%	0.2%
M (11,9) for $t = 1$ month	0.0%	0.0%

S8.1.7. Crystals

Depending on the deposition time of the sample on mica it is possible to cover the entire surface in a similar way to reconfigurable crystals that were studied by simulations in the literature (references in main text) (Figure S9).

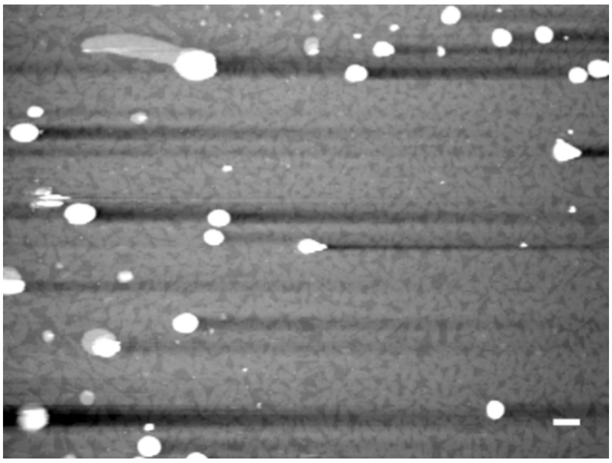

Figure S9. Crystal made of shape-variable monomer M (11,11). Scale bar is 100 nm.

S8.2. After Four Hours Reconfiguration

Experiments under the "reconfiguration protocol" (Supplementary Information S5) were performed. In general, closed structures and larger clusters (more than 6-mer) where observed (Figure S10).

S9. AFM of Fixed Monomers in Narrow Configuration

Fixed monomers in narrow configurations are designed to self-assemble into triangles (Figure S11a). In our case, we only do experiments with M ($a = 11$, $b = 0$).

S9.1. Immobilization on Mica

Figure S11a–b shows the detail of the self-assembled triangles. Broken monomers are also formed, indicating a poor origami folding or the influence of the AFM tip on the observation. Determining whether or not to count them as well-formed monomers is subjective. For instance, the broken monomer in Figure S11b is counted as a component monomer of 3-mer. Figure S9.1c shows a representative AFM image for a deposition time of 1 min. The yield is calculated as described in Supplementary Information S7.

AFM images of the fixed monomer *M* (11,0) indicate as much as a 19.2% yield of trimers in contrast to the "*cis* full-bond yield of 83%" in the original paper of Woo and Rothemund [4]. This difference in yield may be due to three reasons:

- The size of our monomer is similar to the one of Woo; however, the shape of our monomer has an indentation in the outer most corner, which may affect the self-assembly process. Therefore, the edge geometry (shape) of the fixed monomer may affect the self-assembly.
- Due to the long extra M13 that is freely moving outside the monomer; it may interact with colliding monomers and, therefore, affect the assemble dynamics.
- Our experimental protocol differs with that of Woo, in which they diluted 2 μL of sample into 20 μL of buffer on mica (and sometimes dilution is done in test tube). In the case of *M* (11,0), no dilution is done.

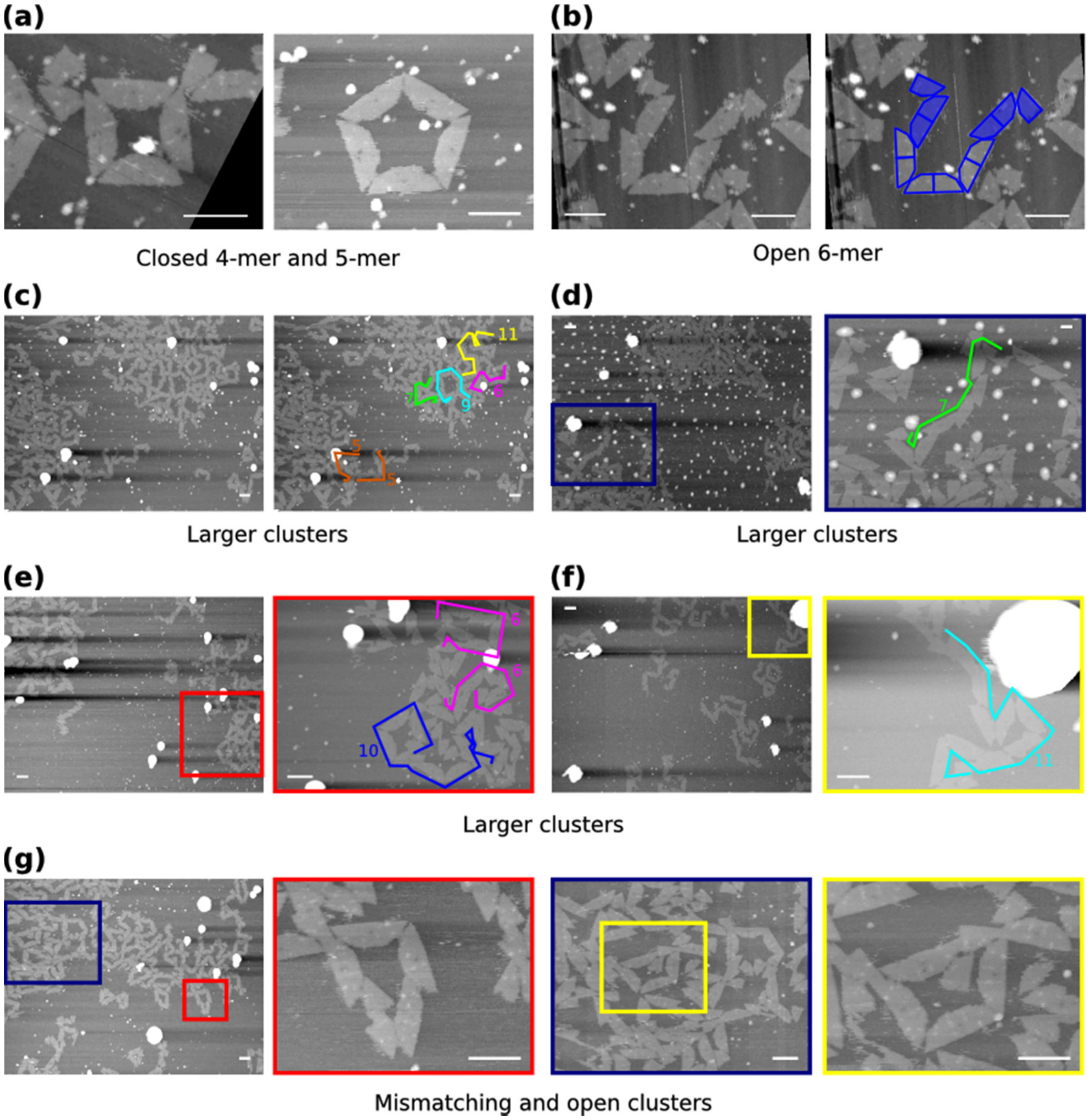

Figure S10. AFM images after 4 h reconfiguration of *M* (11,11).

Instead of diluting, samples are deposited at different times (1 min and 3 min). Dimers in Figure S12 may indicate dis-assembled 3-mers for a deposition time of 1 min. However, considering that DNA

origami may bind strongly and quickly to mica in the presence of Mg^{2+} (structures are unlikely to move under AFM observation), we may say that the distance between dimers is large enough to not represent dimers that belong to broken trimers or larger assemblies. However, detachment and posterior diffusion of monomers is a rare case, as seen in Figure S12. The yields between 1 min (See main text Figure 3a.3 for before reconfiguration) and 3 min are similar (data not shown).

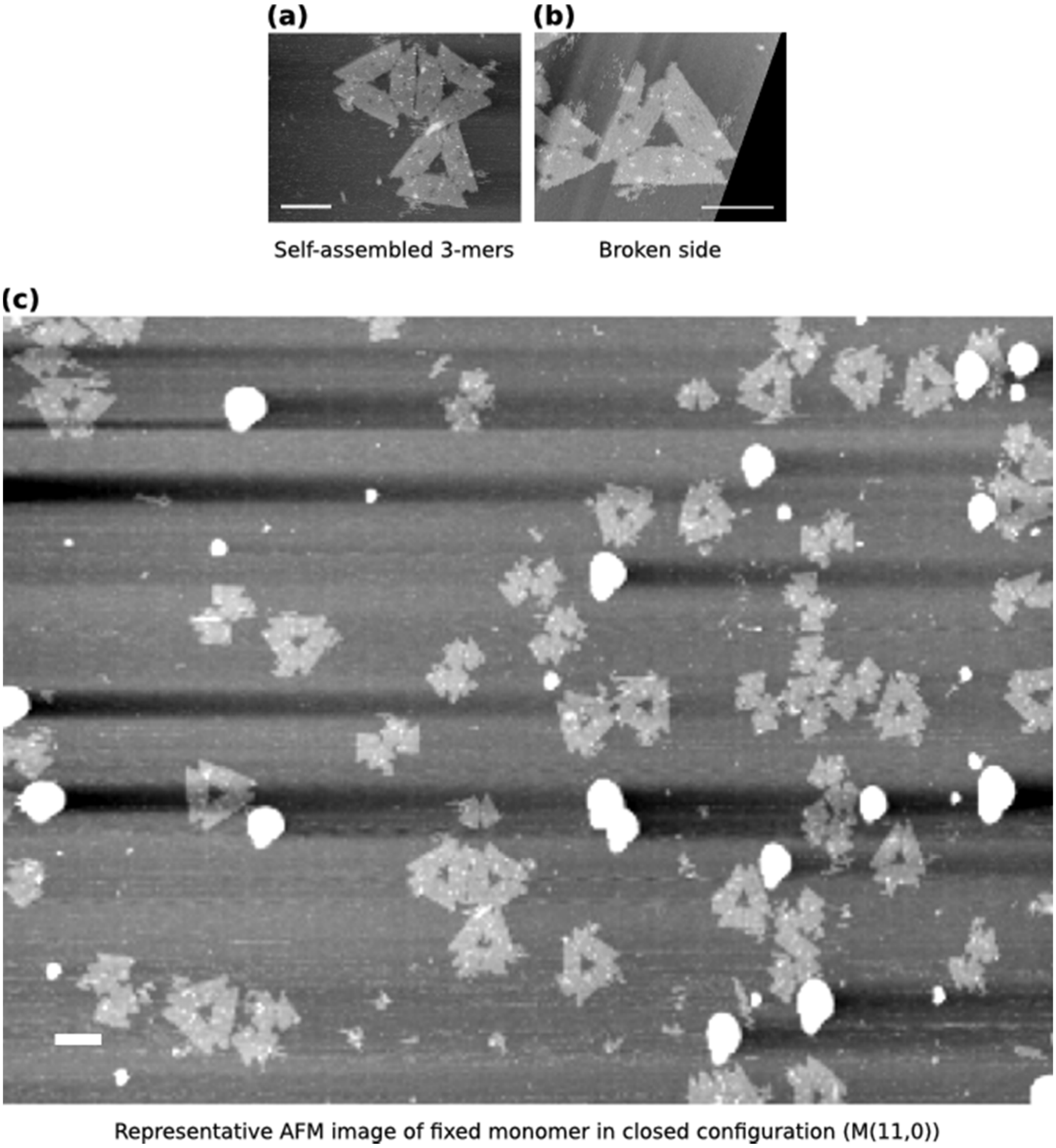

Figure S11. Self-assembled trimers.

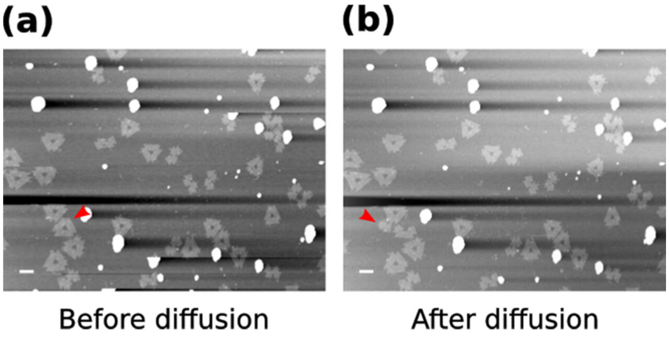

Figure S12. Diffusion of monomer in two consecutive frames under the protocol for AFM observation (S4). Monomer indicated in red (**a**) diffused to a different position (**b**). Later frames showed no posterior movement of the monomers. Scale bars are 100 nm.

Kinetic trapped dimers are formed with dislocated stacking bonds [4]. For example, Figure S13d shows a mismatching in which helices are partially (or fully) shared (compared to stacking due to parallel polarity in Supplementary Information S8.1.3).

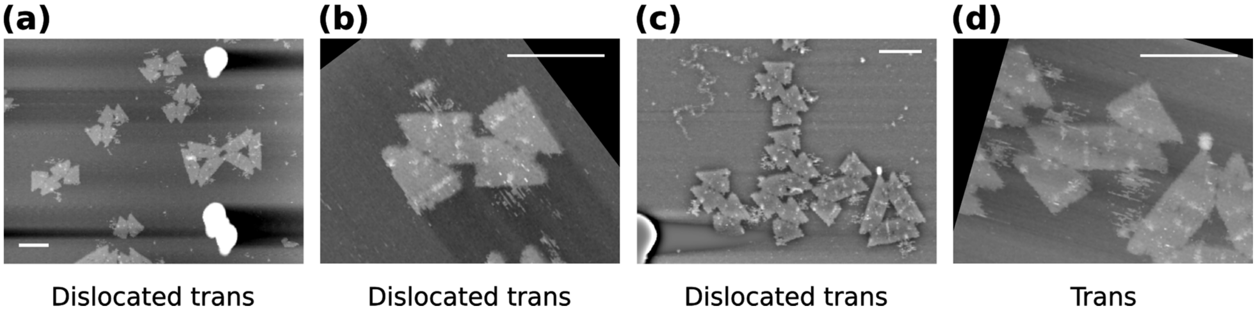

Figure S13. Mismatching: dislocation and *trans* configuration (M (11,0)). Scale bars are 100 nm.

S9.2. Reconfiguration

The yield of the trimers does not change drastically after reconfiguration (data not shown).

S10. AFM of Fixed Monomers in Wide Configuration

Reconfiguration

Fixed monomers M (0,11) on mica are prepared by following the “reconfiguration protocol” (Supplementary Information S6). Nanostructures are then observed by AFM under 1 × TAE/Mg^{2+}. Figure 3b.3 in main text shows a representative image of the nanostructures observed after the “reconfiguration protocol”. In this case, some 6-mers are observed (Figure 3b.12 in main text). We can speculate two causes for the formation of 6-mers.

- There is a geometrical restriction in the movement of the origami such that the structural fluctuation of the monomer is restricted on the surface (compared to fluctuation in free solution) favoring on-plane connections with neighboring monomers.

- The concentration increment of NaCl enhances the electrostatic screening between the arms of the monomer and consequently allowing a wider angle for assembling 6-mers.

S11. Longer Time at Room Temperature (RT) of Sample M (11,9) and M (0,11) before Deposition

Sample *M* (11,9) and *M* (0,11) are observed for different times at RT after annealing: same day of preparation and for more than one month. Figures S14 and S15 shows representative AFM images of samples after annealing ($t = 0$) and after a long time at RT ($t = 1$ month), respectively. Figure S16a,c show the distributions for both *M* (11,9) and *M* (0,11). Additionally, the ratio of open:closed clusters is calculated for both *M* (11,9) and *M* (0,11) in Figure S16b and Figure S16d, respectively. In the case of *M* (11,9), the distribution peak changes from 2-mers to 3-mers, while the number of 4-mers do not vary (Figure S16a). Figure S16b indicates that the ratio closed:open clusters increases for 3-mers ($p < 0.01$, p are shown in Supplementary Information S12.2) and 4-mers ($p < 0.05$) after a long time. In the case of *M* (0,11) the distribution peak changes from 1-mers to 4-mers (Figure S16c). Figure S16d indicates that the ratio closed:open clusters increases for 4-mers ($p < 0.05$) after a long time. We do a simulation based on a chemical kinetics model for the formation of the clusters over time, and find that the tendency in the cluster evolution matches qualitatively with the experimental data. This simulation suggests that the system will reach equilibrium after a longer time. This simulation will be published elsewhere.

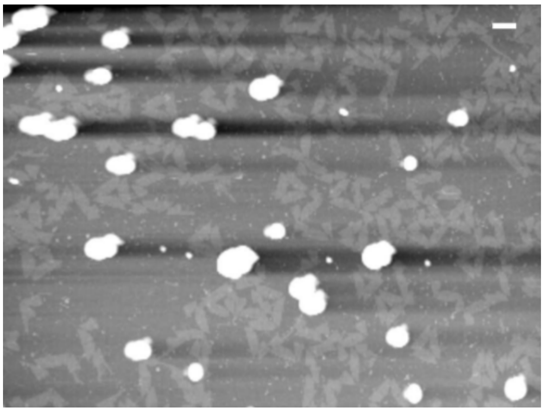

Figure S14. Representative AFM image of the polymorphic clusters made of *M* (11,9). Scale bar is 100 nm.

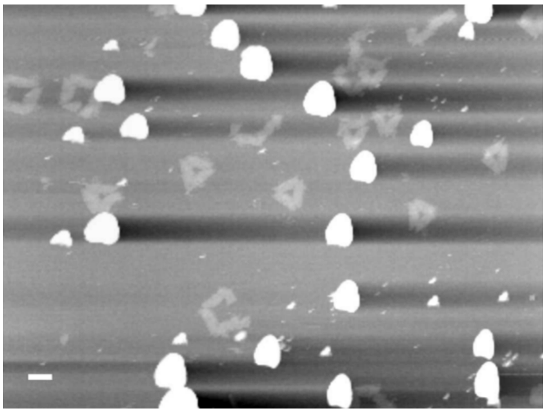

Figure S15. Representative AFM image of the polymorphic clusters made of *M* (11,9) for a longer time at RT after annealing ($t = 1$ month). Scale bar is 100 nm.

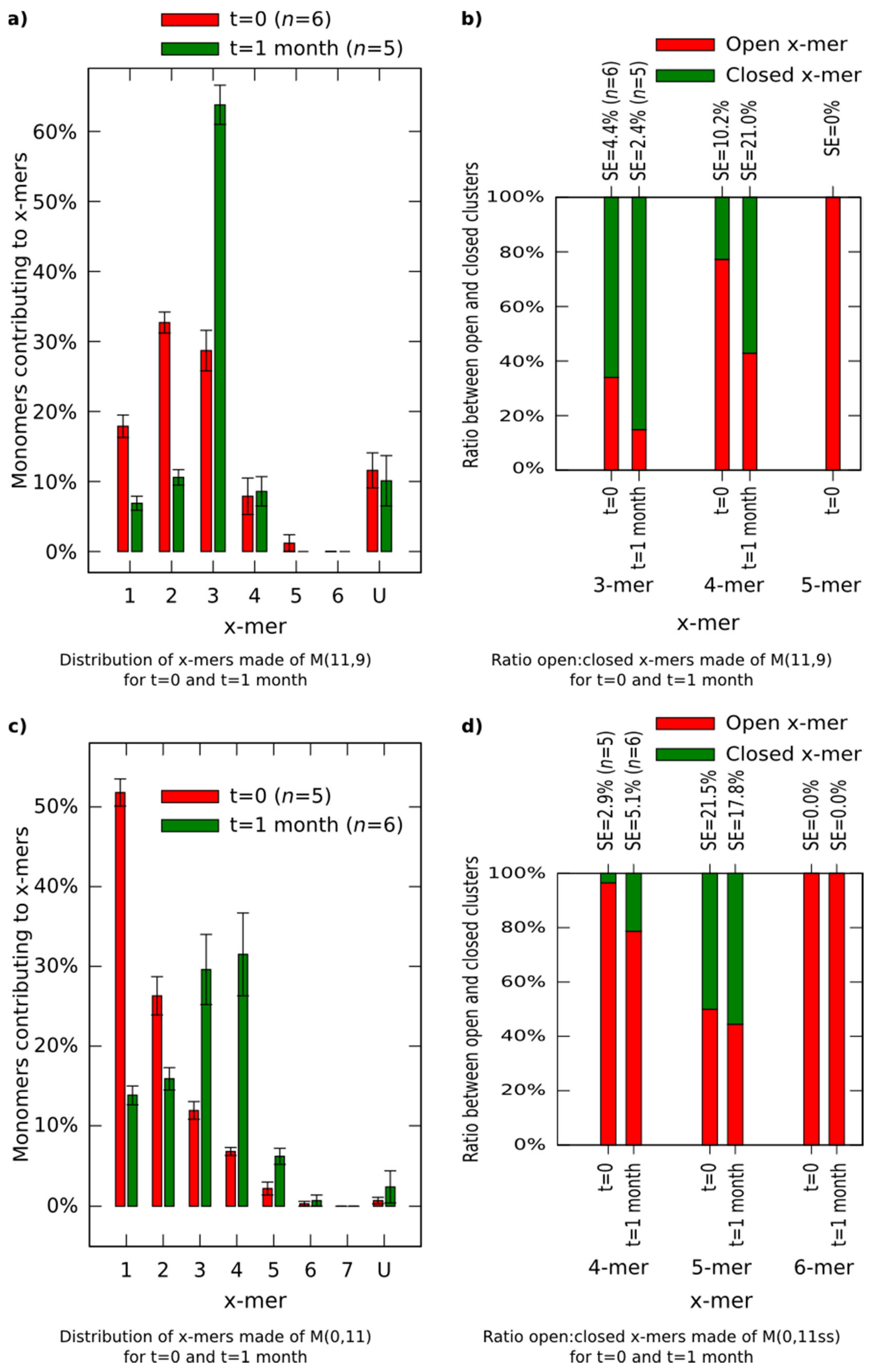

Figure S16. Distributions for *M* (11,9) and *M* (0,11) after annealing (t = 0) and after long time at RT (t = 1 month). (**a**)–(**b**) *M* (11,9). (**c**)–(**d**) *M* (0,11). n indicates the number of analyzed AFM images of 2040 nm × 1680 nm, and the number of counted monomers per each AFM image is shown in S12.

S12. Data Sizes

Number of counted monomers per each AFM image of 2040 nm × 1680 nm is as follows.
Before reconfiguration:

M (0,11)	(334,356,394,394,241).
M (0,11) *t* = 1 month	(93,145,127,148,152,65).
M (11,9)	(209,212,176,172,205,141).
M (11,11)	(122,347,288).
M (11,18)	(93,121,110).
M (11,9) *t* = 1 month	(49,59,81,74,86).
M (11,0)	(61,70).

After reconfiguration:

M (0,8)	(223,304,211,230,157,232,200,271,211,275,251,270,218,156).
M (0,9)	(248,245,339,339,358).
M (0,10)	(227,192,239,179,274,192,178,161).
M (0,11)	(149,488,367,491,400,209,230,183,187).
M (0,12)	(271,358,335,312,276,274,324,285,334).
M (0,13)	(181,175,138,248,185,217,236,218).
M (0,14)	(182,205,155,144).
M (0,15)	(170,131,229,212,147,166,142,168).
M (0,16)	(240,268,277,270,274,281).
M (0,17)	(110,92,250,180,123,145,177,182,168,79).
M (0,18)	(194,226,233,209,208,168).
M (11,0)	(65,109,83,58,64).

S12.1. Standard Error (SE)

Table S4. SE for Monomers contributing to *x*-mers in Figure 4a in the main text.

***M* (0,*b*)*x*-mers**	**1-mers**	**2-mers**	**3-mers**	**4-mers**	**5-mers**	**6-mers**	**7-mers**	**Unclear**
M (0,8)	0.7%	1.6%	1.4%	1.3%	1.0%	0.3%	0.3%	0.3%
M (0,9)	1.8%	2.0%	1.7%	0.8%	0.9%	0.5%	0.8%	0.7%
M (0,10)	2.0%	1.9%	1.8%	1.3%	1.1%	0.9%	0.0%	0.7%
M (0,11)	2.2%	2.0%	1.0%	1.1%	1.0%	0.5%	0.2%	0.3%
M (0,12)	1.6%	1.4%	1.0%	1.2%	1.2%	0.3%	0.0%	0.3%
M (0,13)	1.1%	1.1%	2.0%	1.3%	1.7%	1.0%	0.0%	0.6%
M (0,14)	1.9%	4.5%	1.2%	6.3%	2.0%	2.1%	0.9%	1.1%
M (0,15)	1.4%	1.7%	2.9%	1.4%	1.6%	1.1%	0.8%	0.7%
M (0,16)	1.4%	1.7%	0.9%	2.1%	1.5%	0.7%	0.5%	0.5%
M (0,17)	0.7%	2.1%	2.2%	1.5%	1.8%	0.5%	0.5%	1.2%
M (0,18)	1.7%	2.5%	1.0%	1.4%	1.4%	0.6%	0.0%	0.7%

S12.2. P-Values

For a given x-mer, p-values are calculated for all the possible pairs among the different spring lengths by using a spreadsheet software (libreoffice 4.2.6.3). $P_{bi \to bf}$ indicates the p-value between spring $b = b_i$ and spring $b = b_f$, these springs are called critical springs. Critical springs depict a region $b_i \to b_f$.

The following p-values are all related with Figure 2c in the main text. In bold are those $p < 0.05$.

1-mers
$P_{b = 9 \to b = 11}$ = **0.001**
$P_{b = 9 \to b = 11}$ = **0.001**
$P_{b = 9 \to b = 18}$ = **0.00003**
2-mers
$P_{b = 9 \to b = 11}$ = 0.07
$P_{b = 11 \to b = 18}$ = **0.0089**
$P_{b = 9 \to b = 18}$ = **0.0004**
3-mers
$P_{b = 9 \to b = 11}$ = 0.31
$P_{b = 11 \to b = 18}$ = **0.03**
$P_{b = 9 \to b = 18}$ = **0.0004**
4-mers
$P_{b = 9 \to b = 11}$ = 0.45
$P_{b = 11 \to b = 18}$ = 0.48
$P_{b = 9 \to b = 18}$ = 0.48
5-mers
$P_{b = 9 \to b = 11}$ = 0.46
$P_{b = 11 \to b = 18}$ = 0.497
$P_{b = 9 \to b = 18}$ = 0.46
6-mers
$P_{b = 9 \to b = 11}$ = 0.1
$P_{b = 9 \to b = 18}$ = 0.0935
Unclear
$P_{b = 9 \to b = 11}$ = 0.1
$P_{b = 11 \to b = 18}$ = 0.1132
$P_{b = 9 \to b = 18}$ = 0.2

The gap is the number of error bar arms that would fit between the bottom of the error on one spring length and the top of the error bars on other spring length for a given x-mer [3]. For all the possible pairs between b = 8 nt to b = 18 nt, the lowest p-values satisfying $p < 0.05$, as well as their corresponding gaps, are shown below. p-values are related with Figure 4a in the main text.

1-mer

$\text{gap}_{b = 8 \rightarrow b = 12} = 13.3$	$P_{b = 8 \rightarrow b = 12} = 3.35 \times 10^{-7}$
$\text{gap}_{b = 12 \rightarrow b = 15} = 9.1$	$P_{b = 12 \rightarrow b = 15} = 5.55 \times 10^{-7}$
$\text{gap}_{b = 15 \rightarrow b = 17} = 9.2$	$P_{b = 15 \rightarrow b = 17} = 6.88 \times 10^{-6}$
$\text{gap}_{b = 17 \rightarrow b = 18} = 3.9$	$P_{b = 17 \rightarrow b = 18} = 2.98 \times 10^{-3}$

2-mer

$\text{gap}_{b = 8 \rightarrow b = 13} = 2.0$	$P_{b = 8 \rightarrow b = 13} = 5.43 \times 10^{-3}$
$\text{gap}_{b = 13 \rightarrow b = 15} = 4.0$	$P_{b = 13 \rightarrow b = 15} = 6.98 \times 10^{-4}$
$\text{gap}_{b = 15 \rightarrow b = 18} = 2.3$	$P_{b = 15 \rightarrow b = 18} = 7.56 \times 10^{-3}$

3-mer

$\text{gap}_{b = 8 \rightarrow b = 11} = 7.3$	$P_{b = 8 \rightarrow b = 11} = 1.41 \times 10^{-5}$
$\text{gap}_{b = 11 \rightarrow b = 15} = 3.6$	$P_{b = 11 \rightarrow b = 15} = 3.19 \times 10^{-3}$
$\text{gap}_{b = 15 \rightarrow b = 16} = 2.1$	$P_{b = 15 \rightarrow b = 16} = 0.015$
$\text{gap}_{b = 16 \rightarrow b = 18} = 0.8$	$P_{b = 16 \rightarrow b = 18} = 0.037$

4-mer

$\text{gap}_{b = 8 \rightarrow b = 11} = 8.7$	$P_{b = 8 \rightarrow b = 11} = 1.09 \times 10^{-7}$
$\text{gap}_{b = 11 \rightarrow b = 15} = 5.1$	$P_{b = 11 \rightarrow b = 15} = 1.04 \times 10^{-4}$
$\text{gap}_{b = 11 \rightarrow b = 15} = 1.5$	$P_{b = 15 \rightarrow b = 17} = 9.88 \times 10^{-3}$

5-mer

$\text{gap}_{b = 8 \rightarrow b = 10} = 1.7$	$P_{b = 8 \rightarrow b = 10} = 9.85 \times 10^{-3}$
$\text{gap}_{b = 10 \rightarrow b = 15} = 5.1$	$P_{b = 10 \rightarrow b = 15} = 0.034$

6-mer

$\text{gap}_{b = 8 \rightarrow b = 13} = 1.2$	$P_{b = 8 \rightarrow b = 13} = 0.040$

7-mer

$\text{gap}_{b = 10 \rightarrow b = 15} = 3.1$	$P_{b = 8 \rightarrow b = 15} = 0.019$
$\text{gap}_{b = 15 \rightarrow b = 18} = 3.1$	$P_{b = 15 \rightarrow b = 18} = 0.019$

Unclear

$\text{gap}_{b = 8 \rightarrow b = 11} = 3.8$	$P_{b = 8 \rightarrow b = 11} = 2.97 \times 10^{-4}$
$\text{gap}_{b = 11 \rightarrow b = 16} = 4.8$	$P_{b = 11 \rightarrow b = 16} = 6.39 \times 10^{-4}$

The following p-values are related with Figure 4b in the main text and are all those which are $p < 0.05$.

4-mer

$P_{b = 8 \rightarrow b = 9} = 0.032$
$P_{b = 8 \rightarrow b = 10} = 0.004$
$P_{b = 8 \rightarrow b = 12} = 0.004$
$P_{b = 8 \rightarrow b = 13} = 0.004$
$P_{b = 8 \rightarrow b = 17} = 0.004$
$P_{b = 8 \rightarrow b = 18} = 0.004$
$P_{b = 10 \rightarrow b = 16} = 0.045$
$P_{b = 12 \rightarrow b = 16} = 0.045$
$P_{b = 13 \rightarrow b = 16} = 0.045$
$P_{b = 16 \rightarrow b = 17} = 0.045$
$P_{b = 16 \rightarrow b = 18} = 0.045$

5-mer

$P_{b = 8 \rightarrow b = 17} = 0.0496$

6-mer

$P_{b = 11 \rightarrow b = 15} = 0.03$
$P_{b = 11 \rightarrow b = 17} = 0.03$
$P_{b = 12 \rightarrow b = 13} = 0.04$

The following *p*-values are all related with Figure S16a in Supplementary Information. $p < 0.05$ are in bold.

1-mer
$P_{t = 0 \rightarrow t = 1 \text{ month}}$ = **0.0002**
2-mer
$P_{t = 0 \rightarrow t = 1 \text{ month}}$ = **0.0000007**
3-mer
$P_{t = 0 \rightarrow t = 1 \text{ month}}$ = **0.000006**
4-mer
$P_{t = 0 \rightarrow t = 1 \text{ month}}$ = 0.4
5-mer
$P_{t = 0 \rightarrow t = 1 \text{ month}}$ = 0.2
6-mer
$P_{t = 0 \rightarrow t = 1 \text{ month}}$ = No value
Unclear
$P_{t = 0 \rightarrow t = 1 \text{ month}}$ = 0.4

The following *p*-values are all related with Figure S16b in SI. $P < 0.01$ are in bold.

3-mer
$P_{\text{ratio open:closed}, t = 0 \rightarrow \text{ratio open:closed}, t = 1 \text{ month}}$ = **0.002**
4-mer
$P_{\text{ratio open:closed}, t = 0 \rightarrow \text{ratio open:closed, t} = 1 \text{ month}}$ = 0.025
5-mer
$P_{\text{ratio open:closed}, t = 0 \rightarrow \text{ratio open:closed}, t = 1 \text{ month}}$ = No value
6-mer
$P_{\text{ratio open:closed}, t = 0 \rightarrow \text{ratio open:closed}, t = 1 \text{ month}}$ = No value

The following *p*-values are all related with Figure S16c in SI. $P < 0.01$ are in bold.

1-mer
$P_{t = 0 \rightarrow t = 1 \text{ month}}$ = **0.0000001**
2-mer
$P_{t = 0 \rightarrow t = 1 \text{ month}}$ = **0.004**
3-mer
$P_{t = 0 \rightarrow t = 1 \text{ month}}$ = **0.005**
4-mer
$P_{t = 0 \rightarrow t = 1 \text{ month}}$ = **0.003**
5-mer
$P_{t = 0 \rightarrow t = 1 \text{ month}}$ = 0.006
6-mer
$P_{t = 0 \rightarrow t = 1 \text{ month}}$ = 0.3
7-mer
$P_{t = 0 \rightarrow t = 1 \text{ month}}$ = No value
Unclear
$P_{t = 0 \rightarrow t = 1 \text{ month}}$ = 0.23

The following *p*-values are all related with Figure S16d in SI. $P < 0.05$ are in bold.

4-mer
$P_{\text{ratio open:closed}, t = 0 \rightarrow \text{ratio open:closed}, t = 1 \text{ month}}$ = **0.014**
5-mer
$P_{\text{ratio open:closed}, t = 0 \rightarrow \text{ratio open:closed}, t = 1 \text{ month}}$ = 0.32

6-mer

$P_{\text{ratio open:closed}, t = 0 \rightarrow \text{ratio open:closed}, t = 1 \text{ month}}$ = No value

S13. DNA Origami Design

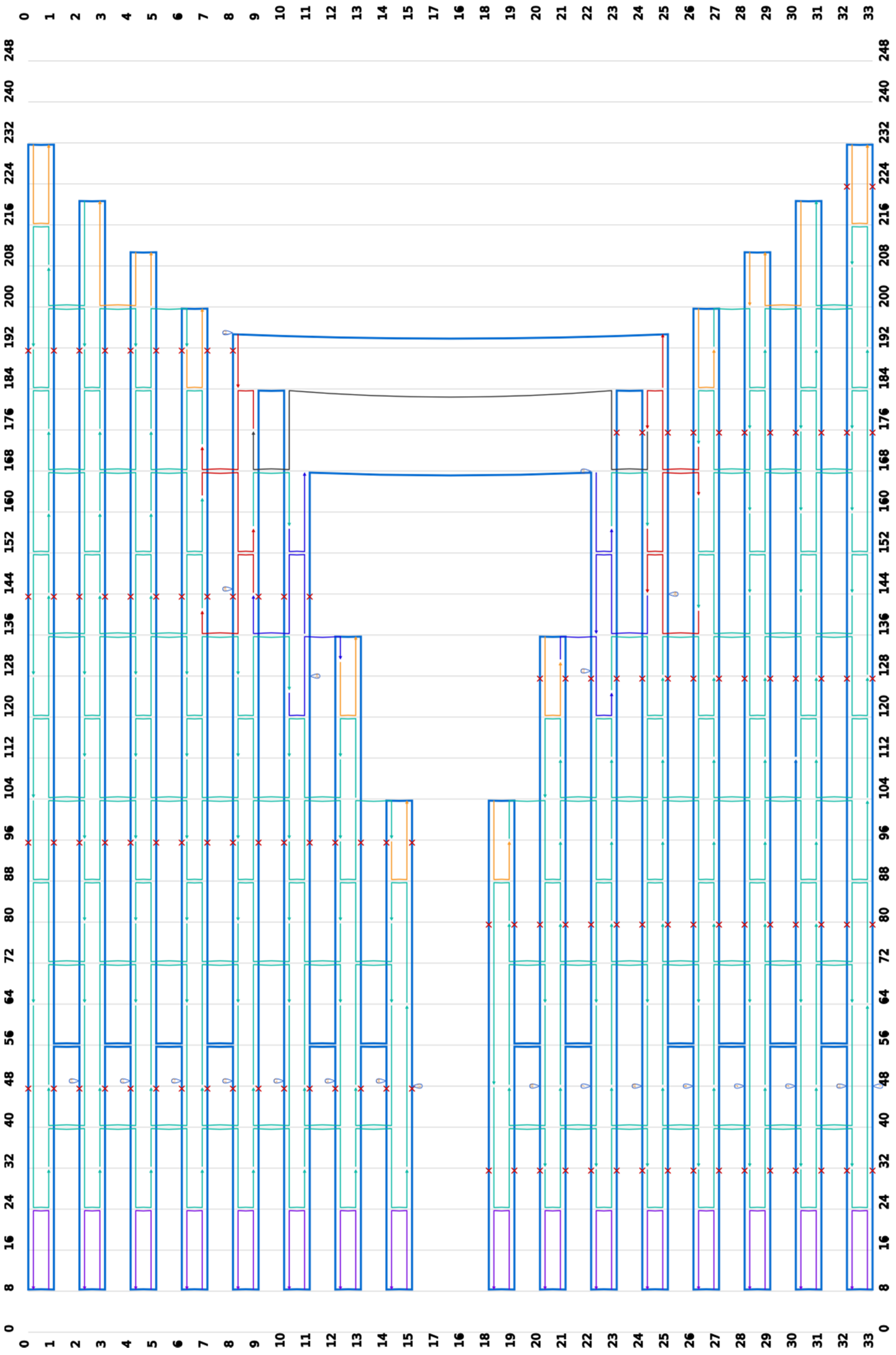

Figure S17. DNA origami design made with cadnano program modified by Woo and Rothemund [4].

SEQUENCES FOR M(11,11)

	Start	End	Sequence	Length
CORE-1	0[63]	1[31]	TACAGGAGTGTACTGGTAATAAGTTTTAACGGGGTCAGTATGCCCCC	47
CORE-2	0[103]	2[96]	TTTACCGTTCCAGTATTCGGTCATAGCCCCCGATAGCA	38
CORE-3	0[127]	2[128]	AGAATGGAATCTTTTCATAATCAAAGCAAGGC	32
CORE-4	0[191]	2[192]	GGTTGAGCCACCCTCAGAACCGTCACCGAC	30
CORE-5	1[32]	3[31]	TGCCTATTTCAAGAGAAGGATTAGTAAGTGCC	32
CORE-6	1[48]	2[64]	CTATTATTGCGTCAGACTGTAGCGATCAAGTT	32
CORE-7	1[144]	0[128]	GGAACCAGACGAATGGATCTTCATTAAAGCC	31
CORE-8	1[160]	3[159]	CCGGAACCCAGCAAAATCACCAGTCGCTAAAG	32
CORE-9	1[176]	1[159]	CAGAGCCGGCAGGTCAGACGATTGGCCTTGATATTCACAAAGCCACCA	48
CORE-10	1[208]	0[192]	AGAGCCACGAGCCGCCGCCAGCATTGACAGGA	32
CORE-11	2[63]	1[47]	TGCCTTTACTGAAACGAGACTCCTCGGAAC	30
CORE-12	2[79]	0[64]	GCGACAGACGTTTTCATCGGCATTAGCGTCATACATGGCTTTTGATGA	48
CORE-13	2[95]	4[96]	GCACCGTAAAGACACCACGGAAATTAAGAC	30
CORE-14	2[111]	0[104]	GAAACCATCTTATTAGCGTTTGCCAAGCGCAGTCTCTGAA	40
CORE-15	2[127]	4[128]	CGGAAACGCAATCAATAGAAAATTATAATAAC	32
CORE-16	2[191]	4[192]	TTGAGCCTGAGGGAGGGAAGGTCTTTTTAA	30
CORE-17	2[220]	1[207]	ATTAAAGGTGAATTATCACCGCCACCCTC	29
CORE-18	3[32]	5[31]	GTCGAGAGCGCCACCCTCAGAACCCACCCTCA	32
CORE-19	3[48]	4[64]	ATAAGTATTACATAAAGGTGGCAAAAACGTAG	32
CORE-20	3[144]	1[143]	TTTACCAGAGCACCATTACCATTAATCACC	30
CORE-21	3[160]	5[159]	ACAAAAGGAAAGTTACCAGAAGGAAAGAATTG	32
CORE-22	3[176]	1[175]	CAACCGATATTTGGGAATTAGAGCGCCTCCCT	32
CORE-23	4[63]	3[47]	AAAATACAAGCCCGGTTTAGTACGGTTGAT	30
CORE-24	4[79]	2[80]	ATGTTAGCCATATAAAAGAAACGCAATCAGTA	32
CORE-25	4[95]	6[96]	TCCTTATAACTGAACACCCTGATTTAACGT	30
CORE-26	4[111]	2[112]	CTGGCATGTAAGTTTATTTTGTCATCACCAAT	32
CORE-27	4[127]	6[128]	GGAATACCATTGAGCGCTAATATCTTATCCCA	32
CORE-28	4[191]	6[192]	GAAAAGTGAAACAATGAAATAGAACGCTAA	30
CORE-29	5[32]	7[31]	TTTTCAGGTTCGTCACCAGTACAACTCATAGT	32
CORE-30	5[48]	6[64]	AGCCCAATAAAAACAGGGAAGCGCACAGAGAG	32
CORE-31	5[144]	3[143]	TAACCCACAACCGAGGAAACGCACATATGG	30
CORE-32	5[160]	7[162]	AGTTAAGCTTGCCAGTTACAAAATTTAGTTGCTAT	35
CORE-33	5[176]	3[175]	AAGAGCAAAAGCAGATAGCCGAACGCGACATT	32
CORE-34	6[63]	5[47]	AATAACATAGGAACCCACTGAGTGATAGCA	30
CORE-35	6[79]	4[80]	CAGCCTTTATTAGACGGGAGAATTTACGCAGT	32
CORE-36	6[95]	8[96]	CAAAAATGAACGCGAGGCGTTTTTTTTATT	30
CORE-37	6[111]	4[112]	ATTTTTTGACAAAGTCAGAGGGTACAAAAGAA	32
CORE-38	6[127]	8[128]	ATCCAAATTTGCGGGAGGTTTTGAAAGTACCG	32
CORE-39	7[32]	9[31]	TAGCGTAAGGGTTTTGCTAAACAAAGGAACAA	32
CORE-40	7[48]	8[64]	AAGTTTTGATCAGATATAGAAGGCGCCCAATA	32
CORE-41	7[141]	5[143]	TAAATCAAGAAAACAGCCATATTATAGAGAGA	32
CORE-42	7[173]	5[175]	GCTACAATTTTCTTTCCAGAGCCTAATCCAATAAT	35
CORE-43	8[63]	7[47]	GCAAGCAATCGTCTTTCTGTATGCGATCTA	30
CORE-44	8[79]	6[80]	ATTACCGCTTATCCGGTATTCTAAGAAAATAG	32
CORE-45	8[95]	10[96]	TTCATCGCTCCAAAAGGAGCCTGTCACCCT	30
CORE-46	8[111]	6[112]	GCAAGCCGTAGCGAACCTCCCGACAAGAAACG	32
CORE-47	8[127]	10[125]	CACTCATCATCAGCTTGCTTTCGATGCAGGGAGTT	35
CORE-48	9[32]	11[31]	CTAAAGGAACTACGAAGGCACCAAAACACTCA	32
CORE-49	9[48]	10[64]	ATAATAATTTTTTCACGTTGAAAAACGAGGGT	32
CORE-50	9[157]	10[157]	TGATACCGATACGCATAACCGA	22
CORE-51	10[63]	9[47]	AGCAACGGCTACAGAGTAATGCCATTGCGA	30
CORE-52	10[79]	8[80]	GCATCGGATCTCCAAAAAAAAAGGTAGGAATC	32
CORE-53	10[95]	12[96]	CAGCAGCATGTTTAGACTGGATCGTTTACC	30
CORE-54	10[111]	8[112]	GCGGGATCTTAATTGTATCGGTTTGAGAACAA	32
CORE-55	11[32]	13[31]	TCTTTGACCCTGCTCCATGTTACTAGGGAACC	32
CORE-56	11[48]	12[64]	GATTATACAAAGAAGTTTTGCCAGGCGAGAGG	32
CORE-57	12[63]	11[47]	CTTTTGCACAAGCGCATCCGCGACCCCAGC	30
CORE-58	12[79]	10[80]	CCAAAATAAGGGGGTAATAGTAAAGAAAGACA	32
CORE-59	12[95]	14[96]	AGACGACATACCACATTCAACTAGAAAAAT	30
CORE-60	12[111]	10[112]	ATAACCCTAGCGTCCAATACTGCGCCGCTTTT	32
CORE-61	13[32]	15[31]	GAACTGACGGATATTCATTACCCATAAGGCTT	32
CORE-62	13[48]	14[64]	GAAAGAGGAGGTAGAAAGATTCATGGAACAAC	32
CORE-63	13[104]	12[112]	AATGCAGATACATAACACACTATC	24
CORE-64	14[63]	13[47]	ATTATTACACAGATGCAAGAACCCAACTTT	30
CORE-65	14[79]	12[80]	GAACTAACCAGTTGAGATTTAGGAGATAAAAA	32
CORE-66	15[32]	15[63]	GCCCTGACGAGAACAAATTACCTTATGCGAT	31
CORE-67	15[64]	14[80]	TTTAAGAACTGGCTCATTATACCAAATAAAAC	32
CORE-68	18[47]	20[32]	GAGCTGAAAAGGTGGCATCAATTATCATACAGGCAAGGTTATGACC	46
CORE-69	19[48]	20[64]	TAGCAAAACAGTTGATTCCCAATTGTTTCATT	32
CORE-70	19[80]	18[48]	CGAGTAGAGGTCAATAACCTGTTTAGCTATATTTTCATTTGGGGCGC	47
CORE-71	19[96]	21[95]	GACCATTATAAATATGCAACTAAACTCCTTTT	32
CORE-72	20[31]	22[32]	CTGTAATAGGATAAAAATTTTTTGTAGGTA	30
CORE-73	20[63]	19[47]	CCATGTAATTAAGCAACAAAAACACAAAGAAT	32
CORE-74	21[48]	22[64]	CATATATTACAGGTCAGGATTAGACCGGAAGC	32
CORE-75	21[80]	19[79]	TTTAATTGGTACGGTGTCTGGAACTGCGAA	30
CORE-76	21[96]	23[95]	GATAAGAGCGTTTTAATTCGAGCTTTTGAGAG	32
CORE-77	21[112]	20[104]	TGCGGATGGTAGCTCAACATGTTT	24
CORE-78	22[31]	24[32]	AAGATTCCACCATCAATATGATGTAGCCAG	30
CORE-79	22[63]	21[47]	AAACTCCATTAAATGCGAGTAATGAGAACCCT	32
CORE-80	23[48]	24[64]	GTTCTAGCTGATAAATTAATGCCGTTGTTAAA	32
CORE-81	23[80]	21[79]	TAGCTATTTCAAAGCGAACCAGAGAGTACC	30
CORE-82	23[96]	25[95]	ATCTACAATTAATATTTTGTTAAAATTTTCGA	32

Figure S18. *Cont.*

CORE-83	23[112]	21[111]	AGGTCATTAAGACTTCAAATATCGGTCATTTT	32
CORE-84	23[125]	25[127]	AGAGTCTGGAATTGTATAAGCAAATACCGACA	32
CORE-85	23[157]	24[157]	CGGTAATCGTATTGATAATCAG	22
CORE-86	24[31]	26[32]	CTTTCATGGATTCTCCGTGGGATGTAGATG	30
CORE-87	24[63]	23[47]	TCAGCTCATTTTTTAAGCCTTCCTATTCAACC	32
CORE-88	25[48]	26[64]	CGGATTGAAACAACGCCAACATGTGAGAATCG	32
CORE-89	25[80]	23[79]	GCAGAGGCATTCGCGTTAAATTTGAGAGGG	30
CORE-90	25[96]	27[95]	GCCAGTAATAAAGCCAACGCTCAAGATGCAAA	32
CORE-91	25[112]	23[111]	TATAAAGTATTTAAATTGTAAACGAGGCTATC	32
CORE-92	25[128]	27[127]	AAAGGTAAATCATATGCGTTATAAGAAAAC	30
CORE-93	26[31]	28[32]	GGCGCATACGACCGTATCGGCCCCGCTTCT	30
CORE-94	26[63]	25[47]	CCATATTTCCGTAATGTACGTTGGACAAACGG	32
CORE-95	26[162]	28[160]	ATAATTACTAGTTCATCTTCTGACCTATTAGAATC	35
CORE-96	27[48]	28[64]	ATCGCACTAGGTTGGGTTATATAATTTTAACC	32
CORE-97	27[80]	25[79]	TAAATGCTCAGTAGGGCTTAATTAATTTAG	30
CORE-98	27[96]	29[95]	TCCAATCGTTATCAAAATCATAGGACCTGAGC	32
CORE-99	27[112]	25[111]	AGAACGCGCAAATTCTTACCAGTATAAGAGAA	32
CORE-100	27[128]	29[127]	TTTTTCAAATTAAGACGCTGAGACAAGAAA	30
CORE-101	27[192]	29[191]	CCGTGTGACTGTAAATCGTCGCTAGTACATAA	32
CORE-102	28[31]	30[32]	GGTGCCGCTGCGCAACTGTTGGCCAGCTGG	30
CORE-103	28[63]	27[47]	TCCGGCTTCCAGCCAGTTCCGGCATCAGGAAG	32
CORE-104	28[143]	26[141]	TAGCTTAGATATATTTTAGTTAATAAAAAGCCTGT	35
CORE-105	28[159]	30[160]	CTTGAAAATTCATTTGAATTACCTAGCTTGAC	32
CORE-106	28[175]	26[173]	TTTTCCCAATTTAATGGTTTGATAAGAATAAA	32
CORE-107	29[48]	30[64]	ATCGGTGCATCGCGCAGAGGCGAAAATACCAA	32
CORE-108	29[80]	27[79]	TTTCAATTTCTGAGAGACTACCTCTATATG	30
CORE-109	29[96]	31[95]	AAAAGAAGGAAACAATAACGGATTGAGTGTTG	32
CORE-110	29[112]	27[111]	ACAAACATAGAGTCAATAGTGAATCAAGACAA	32
CORE-111	29[128]	31[127]	ACAAAATTGAAAGGAAGGGAAGATATTAAA	30
CORE-112	29[192]	31[191]	ATCAATATATCGGAACCCTAAAGGCTACGTGA	32
CORE-113	30[31]	32[32]	CGAAAGGCGCCAGGGTTTTCCCGGTCGACT	30
CORE-114	30[63]	29[47]	GTTACAAAGGGCCTCTCTATTACGGAAGGGCG	32
CORE-115	30[143]	28[144]	CGTGGCGAAATTACATTTAACAATCATAGCGA	32
CORE-116	30[159]	32[160]	GGGGAAAGGCGAAAAACCGTCTATTGCCCTTC	32
CORE-117	30[175]	28[176]	GATTTAGTTTTTAATGGAAACATTAATTAA	30
CORE-118	31[48]	32[64]	CGTTGTAAATAAATCAAAAGAATAATCGGCAA	32
CORE-119	31[80]	29[79]	ATAGGGTTCGCCTGATTGCTTTGTTATTCA	30
CORE-120	31[96]	33[103]	TTCCAGTTGGCGAAAATCCTGTTTGGAAGCATAAAGTGTA	40
CORE-121	31[112]	29[111]	GAGTCCACAAGCGAAACATCGGGAATGATGAA	32
CORE-122	31[128]	33[127]	GAACGTGGAGCAAGCGGTCCACGATGAGTG	30
CORE-123	31[192]	33[191]	ACCATCACCTTTTCACCAGCGAGATGTCGTGC	32
CORE-124	32[31]	33[63]	CTAGAGGTGGTCATAGCTGTTTCCTGTGTGAATTGTTATCCGCTCA	46
CORE-125	32[63]	31[47]	AATCCCTTAACGACGGTGCCTGCAAGTCACGA	32
CORE-126	32[143]	30[144]	AGAGTTGCACTCCAACGTCAAAGGCCGGCGAA	32
CORE-127	32[159]	32[176]	ACCGCCTGCGCTCACTGCCCGCTTTCCAGTCGGGAAACCCGGGCAAC	47
CORE-128	32[175]	30[176]	AGCTGATCAGGGCGATGGCCCAGAGCCCCC	30
CORE-129	32[207]	31[220]	TGGTTTTTCCAAATCAAGTTTTTTGGGGT	29
CORE-130	33[64]	31[79]	CAATTCCACACAACATACGAGCCGATGGTGGTTCCGAAGCCCGAG	45
CORE-131	33[104]	31[111]	AAGCCTGGGGTGCCTACTGGTTTGCCCCAGCATGGAACAA	40
CORE-132	33[128]	32[144]	AGCTAACTCACATTAATTGCGTTGGCCCTGAG	32
CORE-133	33[192]	32[208]	CAGCTGCATTAATGAATCGGCCAACGCCAGGG	32
CODE-1	1[8]	0[8]	CCCGTATAAACAGTTAGCCTTGAGTAACAGTG	32
CODE-2	3[8]	2[8]	CTCAGTACCAGGCGGAGATTAGCGGGGTTTTG	32
CODE-3 - excluded	5[8]	4[8]	CCACCCTCAGAGCCACGCCACCCTCAGAACCG	32
CODE-4 - excluded	7[8]	6[8]	CATTCCACAGACAACCACTACAACGCCTGTAG	32
CODE-5	9[8]	8[8]	CGGAGTGAGAATAGAACTTTCAACAGTTTCAG	32
CODE-6	11[8]	10[8]	CGAAAGAATACACTAACCTAAAACGAAAGAGG	32
CODE-7	13[8]	12[8]	CAGACGGTCAATCATATAGCCGGAACGAGGCG	32
CODE-8	15[8]	14[8]	CTGCTCATTCAGTGAAAATCAACGTAACAAAG	32
CODE-9	19[8]	18[8]	CATTAACATCCAATAACTACTAATAGTAGTAG	32
CODE-10	21[8]	20[8]	CCTTTATTTCAACGCAACTTTTGCGGGAGAAG	32
CODE-11	23[8]	22[8]	CCGGAGACAGTCAAATAAAAGGGTGAGAAAGG	32
CODE-12	25[8]	24[8]	CGAGTAACAACCCGTCCAACATTAAATGTGAG	32
CODE-13 - excluded	27[8]	26[8]	CCAGTTTGAGGGGACGCGTAACCGTGCATCTG	32
CODE-14 - excluded	29[8]	28[8]	CCATTCGCCATTCAGGGAAACCAGGCAAAGCG	32
CODE-15	31[8]	30[8]	CGATTAAGTTGGGTAAGGGATGTGCTGCAAGG	32
CODE-16	33[8]	32[8]	CTCGAATTCGTAATCAATCCCCGGGTACCGAG	32
HINGE	24[175]	9[175]	ACCCCGGAAACTAGCATGTCAACAACAACCATCGCCCAGTTGCGCC	46
EDGE-A	0[231]	1[231]	ACCAGAACCACCACCACACCCTCAGAGCCGCC	32
EDGE-A	4[210]	3[220]	TTACCGAAGCCAAATATTGACGGAAATTATTC	32
EDGE-A	5[200]	5[210]	CAATAGCTATC	11
EDGE-A	6[191]	7[199]	CGAGCGTATCCTGAATCTTACC	22
EDGE-A	12[130]	13[135]	TAGTAAGAGCAGCCAAAAGGAATTACG	27
EDGE-A	14[95]	15[103]	CTACGTTGTCAGGACGTTGGGA	22
EDGE-B	18[103]	19[95]	GATACATTTCGCAAATTTTAGTTT	24
EDGE-B	20[135]	21[130]	GCTGAATCTGGTGCTGCTTAGAGCT	25
EDGE-B	26[199]	27[191]	TAAATAAGGCGTTAAAAATACCGA	24
EDGE-B	28[210]	28[200]	TAACCTTGCTT	11
EDGE-B	30[220]	29[210]	CGAGGTGCCGTAAAGCACTAAATGTGAGTGAA	32
EDGE-B	32[231]	33[231]	GGTTTGCGTATTGGGCGCGCGGGGAGAGGC	30

Figure S18. *Cont.*

SPRING-A11	7[163]	9[156]	TTTGCAGAAACCAATCAATAACAGCT	26
SPRING-A11	8[194]	8[184]	TATCCCATCC	10
SPRING-A11	9[144]	7[140]	TTCTTAAATCGGCTGATTAAACCAGCCT	28
SPRING-A11	9[176]	7[172]	GACAATGATAATTTACGAGCATGTACCCA	29
SPRING-A11	24[156]	26[163]	AAAAGTGCAGAACGCGCCTGTGAATC	26
SPRING-A11	25[184]	25[194]	AAGTCCTGAAC	11
SPRING-A11	26[140]	24[144]	TTAGTAGTAATTCTCAGCTAACCCCAAAA	29
SPRING-A11	26[172]	24[176]	CACCGTTATCAACAATAGATTCATATGT	28
SPRING-B9	10[124]	12[131]	AAAGGGAATCGTCAATGCTTTAGGCA	26
SPRING-B9	10[156]	11[167]	TATATCGAGAATGACCATAAA	21
SPRING-B9	11[136]	9[143]	AAACAGTTCAGAAAATCGGTCGCTGAGGCTGGTGAAT	37
SPRING-B9	21[131]	23[124]	TAATTAAAGCGGAAGCCCGAGCCTG	25
SPRING-B9	22[167]	23[156]	AGGTCTTTACCCTGACATGAA	21
SPRING-B9	24[143]	22[136]	ACAGGAAGGCAAACAAGAGAATCGTATTATAGTCAGAAGC	40

FOR FIXED MONOMERS

FIXING-EDGE-A	0[231]	33[231]	ACCAGAACCACCACCACACCCTCAGAGCCGCCGGTTTGCGTATTGGGCGCGCGGGGAGAGGC	62
FIXING-EDGE-A	4[210]	29[210]	TTACCGAAGCCAAATATTGACGGAAATTATTCCGAGGTGCCGTAAAGCACTAAATGTGAGTGAA	64
FIXING-EDGE-A	5[200]	28[200]	CAATAGCTATCTAACCTTGCTT	22
FIXING-EDGE-A	6[191]	27[191]	CGAGCGTATCCTGAATCTTACCTAAATAAGGCGTTAAAAATACCGA	46
FIXING-EDGE-A11	25[184]	8[184]	AAGTCCTGAACTATCCCATCC	21
FIXING-EDGE-B	12[130]	21[130]	TAGTAAGAGCAGCCAAAAGGAATTACGGCTGAATCTGGTGCTGCTTAGAGCT	52
FIXING-EDGE-B	14[95]	19[95]	CTACGTTGTCAGGACGTTGGGAGATACATTTCGCAAATTTTAGTTT	46
FIXING-EDGE-B9	10[156]	23[156]	TATATCGAGAATGACCATAAAAGGTCTTTACCCTGACATGAA	42
SPRING-B8	10[124]	12[131]	AAAGGGAATCGTCAATGCTTTAGGCA	26
SPRING-B8	10[156]	11[167]	TATATCGAGAATGACCATAAA	21
SPRING-B8	11[136]	9[143]	AAACAGTTCAGAAAATCGGTCGCTGAGGCTGGTGAAT	37
SPRING-B8	21[131]	23[124]	TAATTCAAAGCGAAGCCCGAGCCTG	25
SPRING-B8	22[167]	23[156]	CAGGTCTTTACCCTGAATGAA	21
SPRING-B8	24[143]	22[136]	ACAGGAAGGCAAACAAGAGAATCGCTATTATAGTCAGAAG	40
SPRING-B10	10[124]	12[131]	AAAGGGAATCGTCAAATGCTTAGGCA	26
SPRING-B10	10[156]	11[167]	TATATACGAGAATGACCATAA	21
SPRING-B10	11[136]	9[143]	TAAACAGTTCAGAAATCGGTCGCTGAGGCTGGTGAAT	37
SPRING-B10	21[131]	23[124]	TAATTAAAGCGGAAGCCCGAGCCTG	25
SPRING-B10	22[167]	23[156]	AGGTCTTTACCCTGACATGAA	21
SPRING-B10	24[143]	22[136]	ACAGGAAGGCAAACAAGAGAATCGTATTATAGTCAGAAGC	40
SPRING-B11	10[124]	12[131]	AAAGGGAATCGTCAAATGCTTAGGCA	26
SPRING-B11	10[156]	11[167]	TATATACGAGAATGACCATAA	21
SPRING-B11	11[136]	9[143]	TAAACAGTTCAGAAATCGGTCGCTGAGGCTGGTGAAT	37
SPRING-B11	21[131]	23[124]	TAATTAAGCGGAAAGCCCGAGCCTG	25
SPRING-B11	22[167]	23[156]	GGTCTTTACCCTGACTATGAA	21
SPRING-B11	24[143]	22[136]	ACAGGAAGGCAAACAAGAGAATCGATTATAGTCAGAAGCA	40
SPRING-B12	10[124]	12[131]	AAAGGGAATCGTCCAAATGCTAGGCA	26
SPRING-B12	10[156]	11[167]	TATATAACGAGAATGACCATA	21
SPRING-B12	11[136]	9[143]	TTAAACAGTTCAGAATCGGTCGCTGAGGCTGGTGAAT	37
SPRING-B12	21[131]	23[124]	TAATTAAGCGGAAAGCCCGAGCCTG	25
SPRING-B12	22[167]	23[156]	GGTCTTTACCCTGACTATGAA	21
SPRING-B12	24[143]	22[136]	ACAGGAAGGCAAACAAGAGAATCGATTATAGTCAGAAGCA	40
SPRING-B13	10[124]	12[131]	AAAGGGAATCGTCCAAATGCTAGGCA	26
SPRING-B13	10[156]	11[167]	TATATAACGAGAATGACCATA	21
SPRING-B13	11[136]	9[143]	TTAAACAGTTCAGAATCGGTCGCTGAGGCTGGTGAAT	37
SPRING-B13	21[131]	23[124]	TAATTAGCGGATAAGCCCGAGCCTG	25
SPRING-B13	22[167]	23[156]	GTCTTTACCCTGACTAATGAA	21
SPRING-B13	24[143]	22[136]	ACAGGAAGGCAAACAAGAGAATCGTTATAGTCAGAAGCAA	40
SPRING-B14	10[124]	12[131]	AAAGGGAATCGTCTCAAATGCAGGCA	26
SPRING-B14	10[156]	11[167]	TATATAAACGAGAATGACCAT	21
SPRING-B14	11[136]	9[143]	TTTAAACAGTTCAGATCGGTCGCTGAGGCTGGTGAAT	37
SPRING-B14	21[131]	23[124]	TAATTAGCGGATAAGCCCGAGCCTG	25
SPRING-B14	22[167]	23[156]	GTCTTTACCCTGACTAATGAA	21
SPRING-B14	24[143]	22[136]	ACAGGAAGGCAAACAAGAGAATCGTTATAGTCAGAAGCAA	40
SPRING-B15	10[124]	12[131]	AAAGGGAATCGTCTCAAATGCAGGCA	26
SPRING-B15	10[156]	11[167]	TATATAAACGAGAATGACCAT	21
SPRING-B15	11[136]	9[143]	TTTAAACAGTTCAGATCGGTCGCTGAGGCTGGTGAAT	37
SPRING-B15	21[131]	23[124]	TAATTGCGGATTAAGCCCGAGCCTG	25
SPRING-B15	22[167]	23[156]	TCTTTACCCTGACTATATGAA	21
SPRING-B15	24[143]	22[136]	ACAGGAAGGCAAACAAGAGAATCGTATAGTCAGAAGCAAA	40
SPRING-B16	10[124]	12[131]	AAAGGGAATCGTCCTCAAATGAGGCA	26
SPRING-B16	10[156]	11[167]	TATATAAAACGAGAATGACCA	21
SPRING-B16	11[136]	9[143]	CTTTAAACAGTTCAGTCGGTCGCTGAGGCTGGTGAAT	37
SPRING-B16	21[131]	23[124]	TAATTGCGGATTAAGCCCGAGCCTG	25
SPRING-B16	22[167]	23[156]	TCTTTACCCTGACTATATGAA	21
SPRING-B16	24[143]	22[136]	ACAGGAAGGCAAACAAGAGAATCGTATAGTCAGAAGCAAA	40

Figure S18. *Cont.*

SPRING-B17	10[124]	12[131]	AAAGGGAATCGTCCTCAAATGAGGCA	26
SPRING-B17	10[156]	11[167]	TATATAAAACGAGAATGACCA	21
SPRING-B17	11[136]	9[143]	CTTTAAACAGTTCAGTCGGTCGCTGAGGCTGGTGAAT	37
SPRING-B17	21[131]	23[124]	TAATTCGGATTGAAGCCCGAGCCTG	25
SPRING-B17	22[167]	23[156]	CTTTACCCTGACTATTATGAA	21
SPRING-B17	24[143]	22[136]	ACAGGAAGGCAAACAAGAGAATCGATAGTCAGAAGCAAAG	40

SPRING-B18	10[124]	12[131]	AAAGGGAATCGTCCCTCAAATAGGCA	26
SPRING-B18	10[156]	11[167]	TATATGAAAACGAGAATGACC	21
SPRING-B18	11[136]	9[143]	GCTTTAAACAGTTCATCGGTCGCTGAGGCTGGTGAAT	37
SPRING-B18	21[131]	23[124]	TAATTCGGATTGAAGCCCGAGCCTG	25
SPRING-B18	22[167]	23[156]	CTTTACCCTGACTATTATGAA	21
SPRING-B18	24[143]	22[136]	ACAGGAAGGCAAACAAGAGAATCGATAGTCAGAAGCAAAG	40

Figure S18. DNA origami sequences used in this work.

References

1. Smith, S.B.; Cui, Y.; Bustamante, C. Overstretching B-DNA: The elastic response of individual double-stranded and single-stranded DNA molecules. *Science* **1996**, *271*, 795–799.
2. Saenger, W. *Principles of Nucleic Acid Structure*; Springer-Verlag: Berlin, Germany, 1984.
3. Cumming, G.; Fidler, F.; Vaux, D.L. Error bars in experimental biology. *J. Cell. Biol.* **2007**, 177, 7–11.
4. Woo, S.; Rothemund, P.W.K. Programmable molecular recognition based on the geometry of DNA nanostructures. *Nat. Chem.* **2011**, *3*, 620–627.

© 2015 by the authors; licensee MDPI, Basel, Switzerland. This article is an open access article distributed under the terms and conditions of the Creative Commons Attribution license (http://creativecommons.org/licenses/by/4.0/).
